# Supplementary material for: Engineering PEG10-assembled endogenous virus-like particles with genetically encoded neoantigen peptides for cancer vaccination
Source: eLife. 2024 Sep 13;13:RP98579. doi: 10.7554/eLife.98579 (PMC11398863; doi:10.7554/eLife.98579)
Supplement: Figure 1—source data 2. [file elife-98579-fig1-data2.pdf]

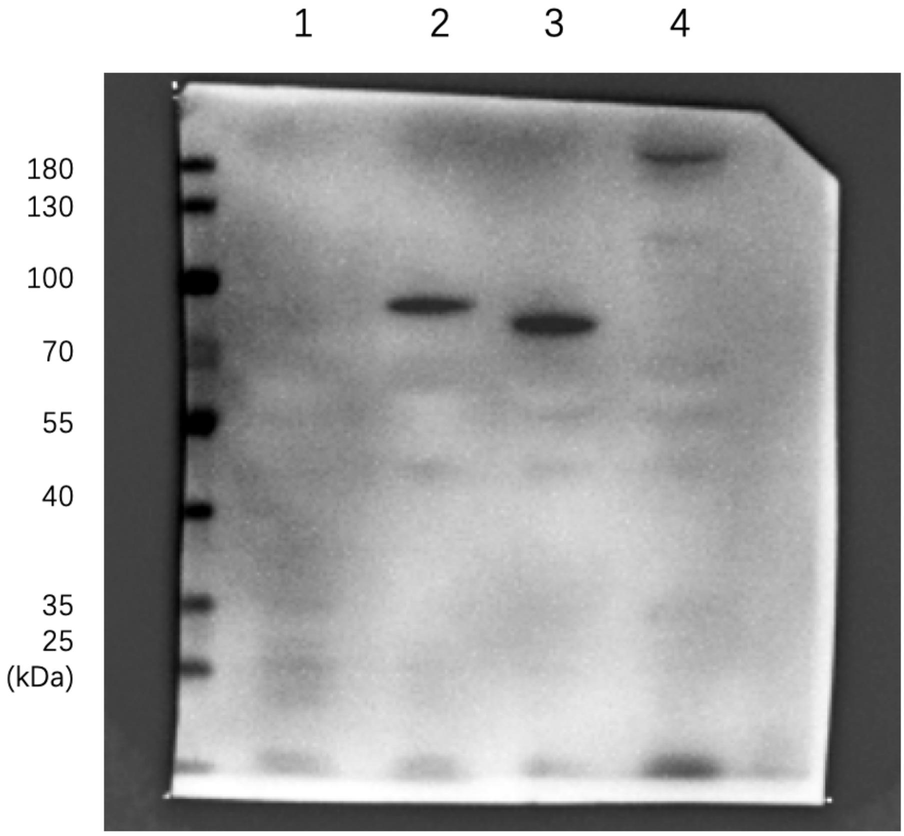

IB: HA tag

1. eGFP + VSVg
2. gag-//-eGFP + VSVg
3. gag-GS-eGFP + VSVg
4. gag-//-pol-eGFP + VSVg

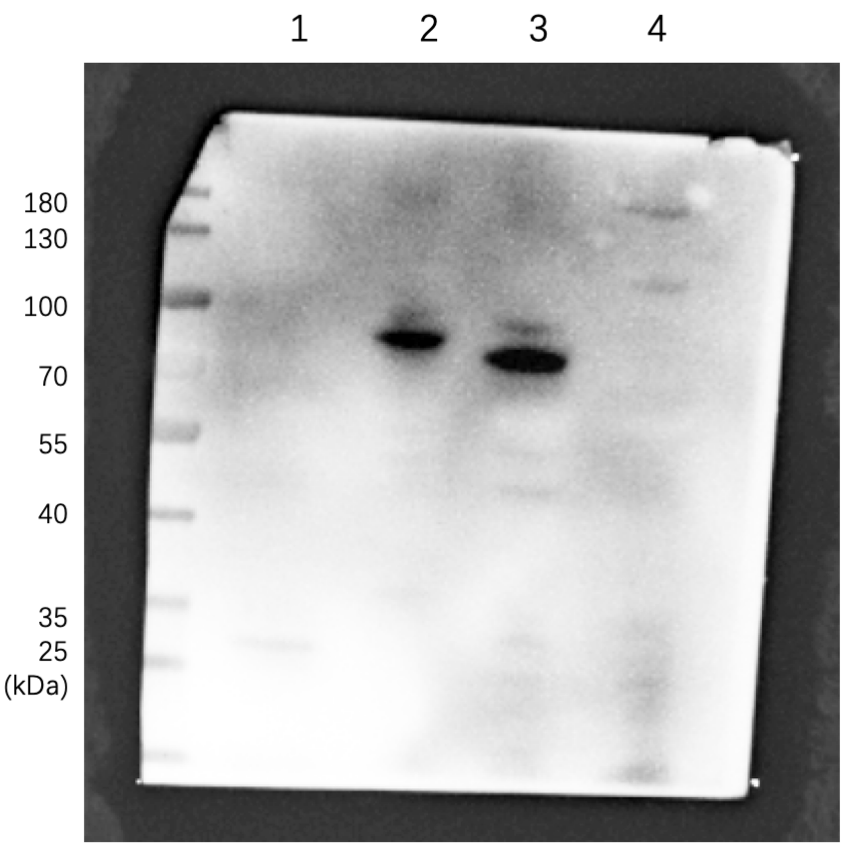

IB: eGFP

1. eGFP + VSVg
2. gag-//-eGFP + VSVg
3. gag-GS-eGFP + VSVg
4. gag-//-pol-eGFP + VSVg
