# Supplementary figures and images for: Engineering PEG10-assembled endogenous virus-like particles with genetically encoded neoantigen peptides for cancer vaccination
Source: eLife. 2024 Sep 13;13:RP98579. doi: 10.7554/eLife.98579 (PMC11398863; doi:10.7554/eLife.98579)

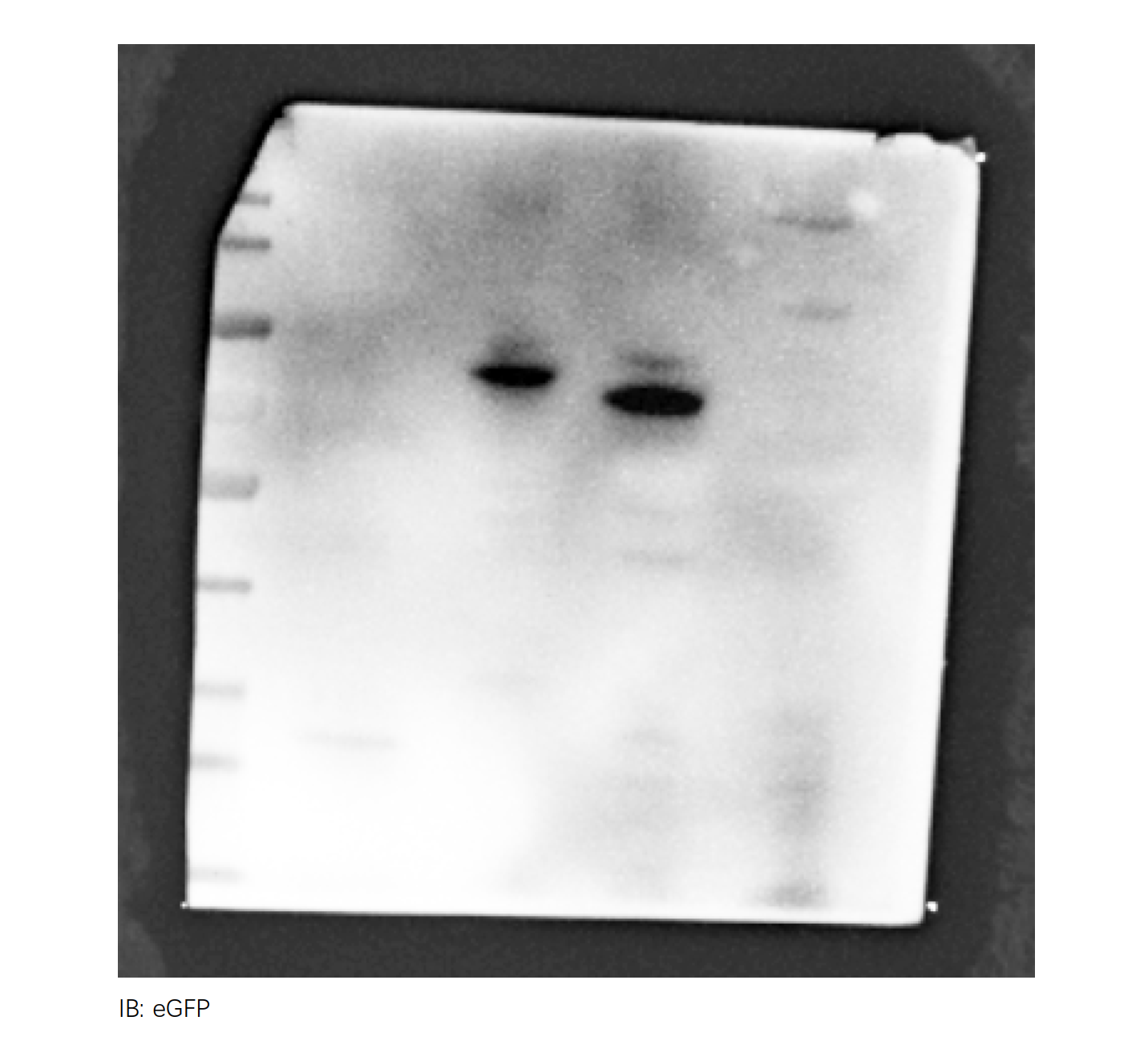

Supplement: Figure 1—source data 3. [file elife-98579-fig1-data3.zip › Figure 1-source data 3/IB eGFP.tif]

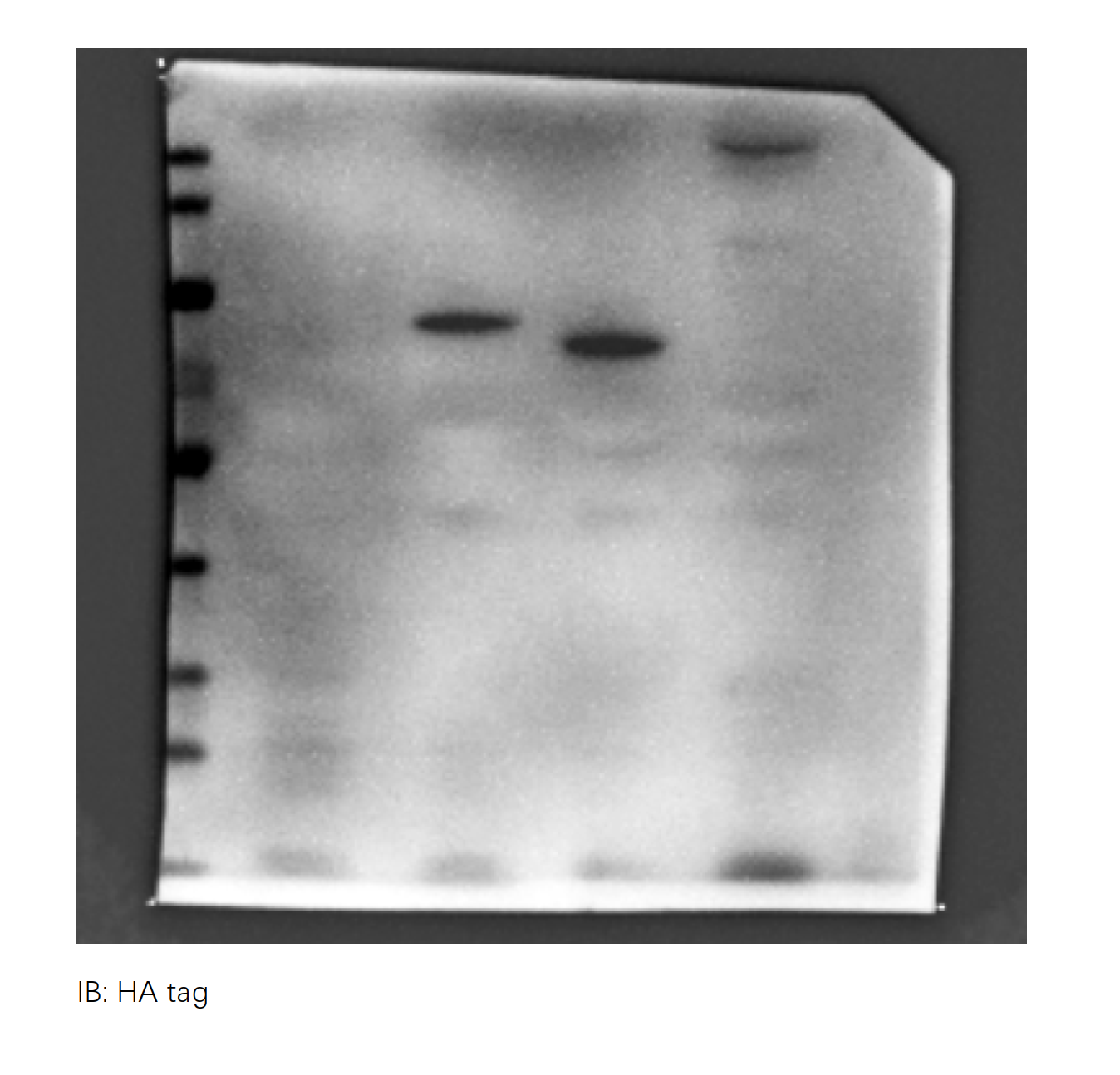

Supplement: Figure 1—source data 3. [file elife-98579-fig1-data3.zip › Figure 1-source data 3/IB HA.tif]

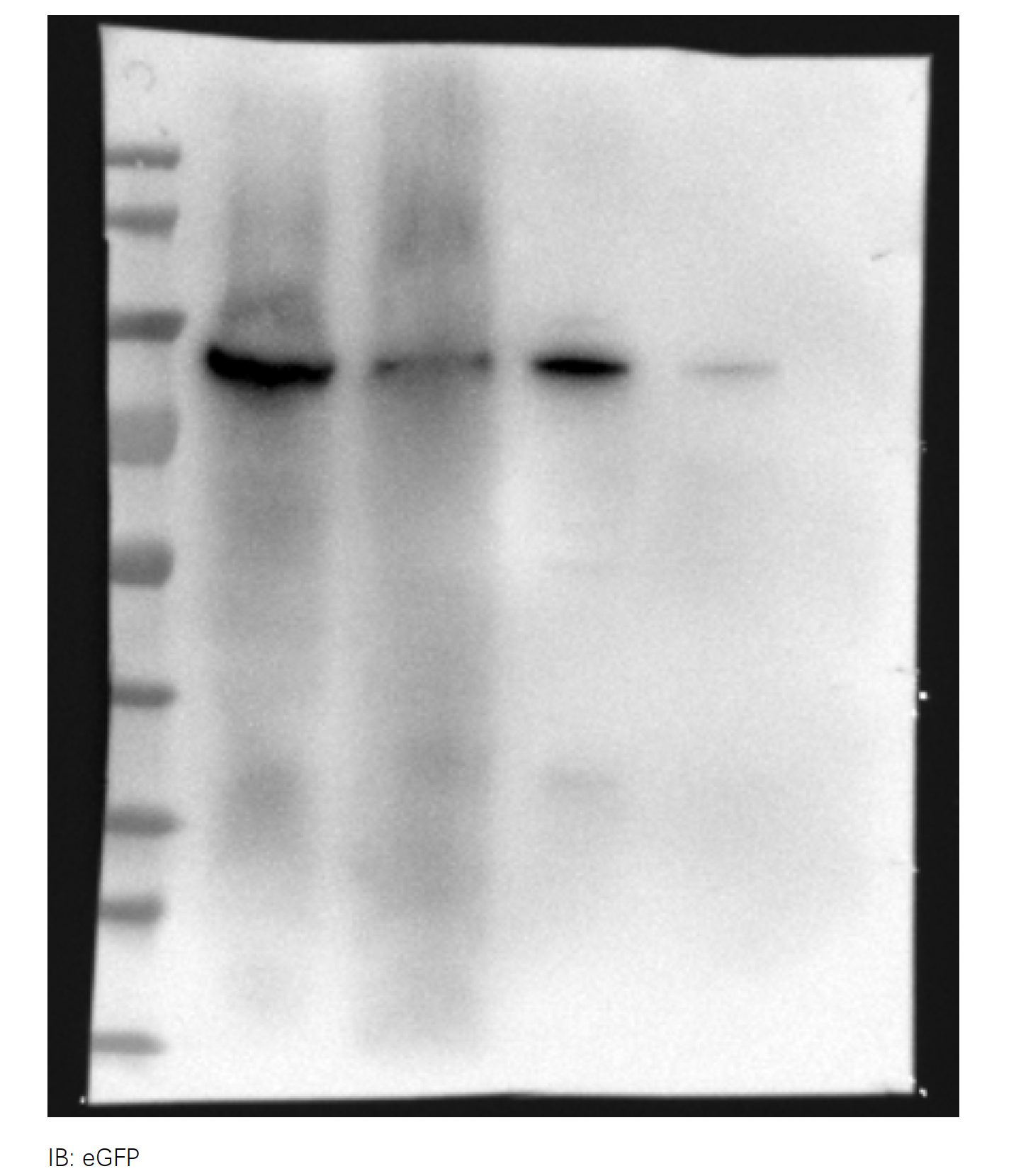

Supplement: Figure 1—figure supplement 1—source data 2. [file elife-98579-fig1-figsupp1-data2.zip › Figure 1-figure supplement 1-source data 2/IB eGFP.tif]

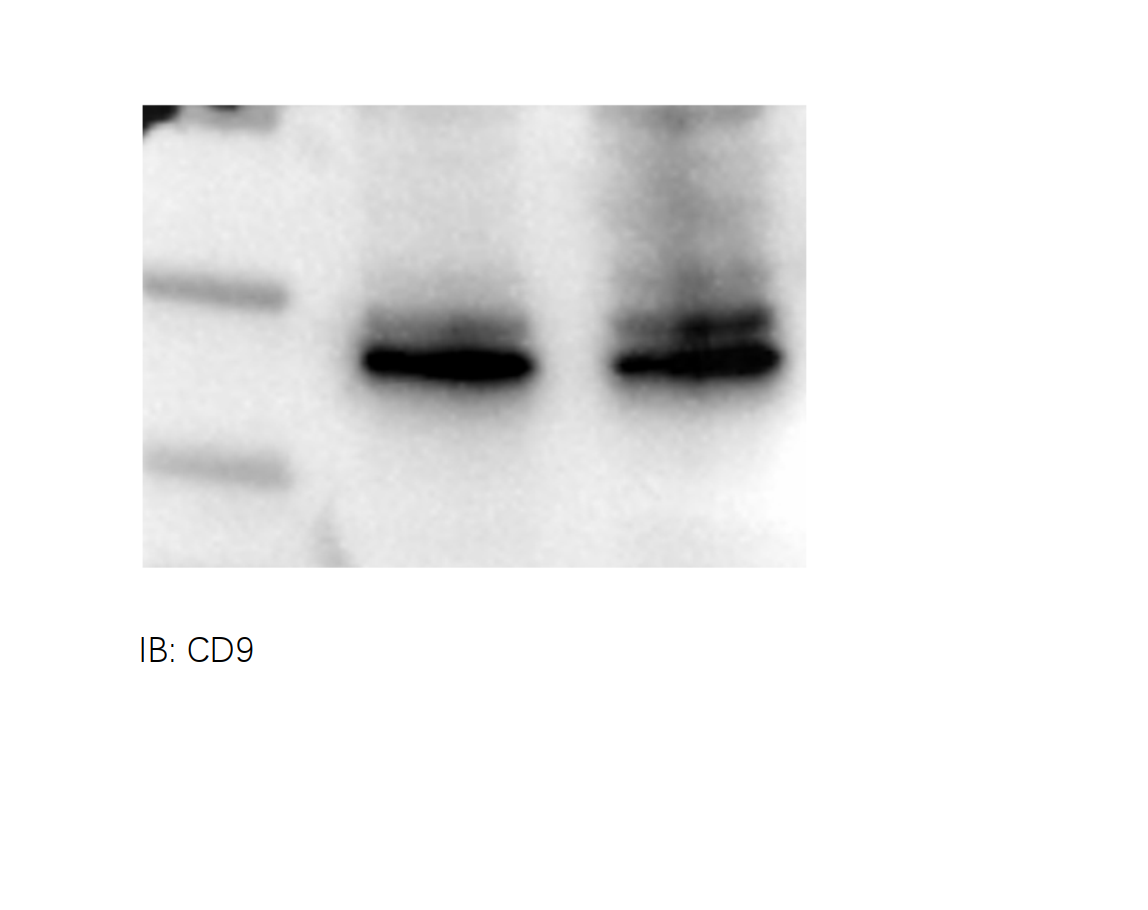

Supplement: Figure 1—figure supplement 1—source data 2. [file elife-98579-fig1-figsupp1-data2.zip › Figure 1-figure supplement 1-source data 2/IB CD9.tif]

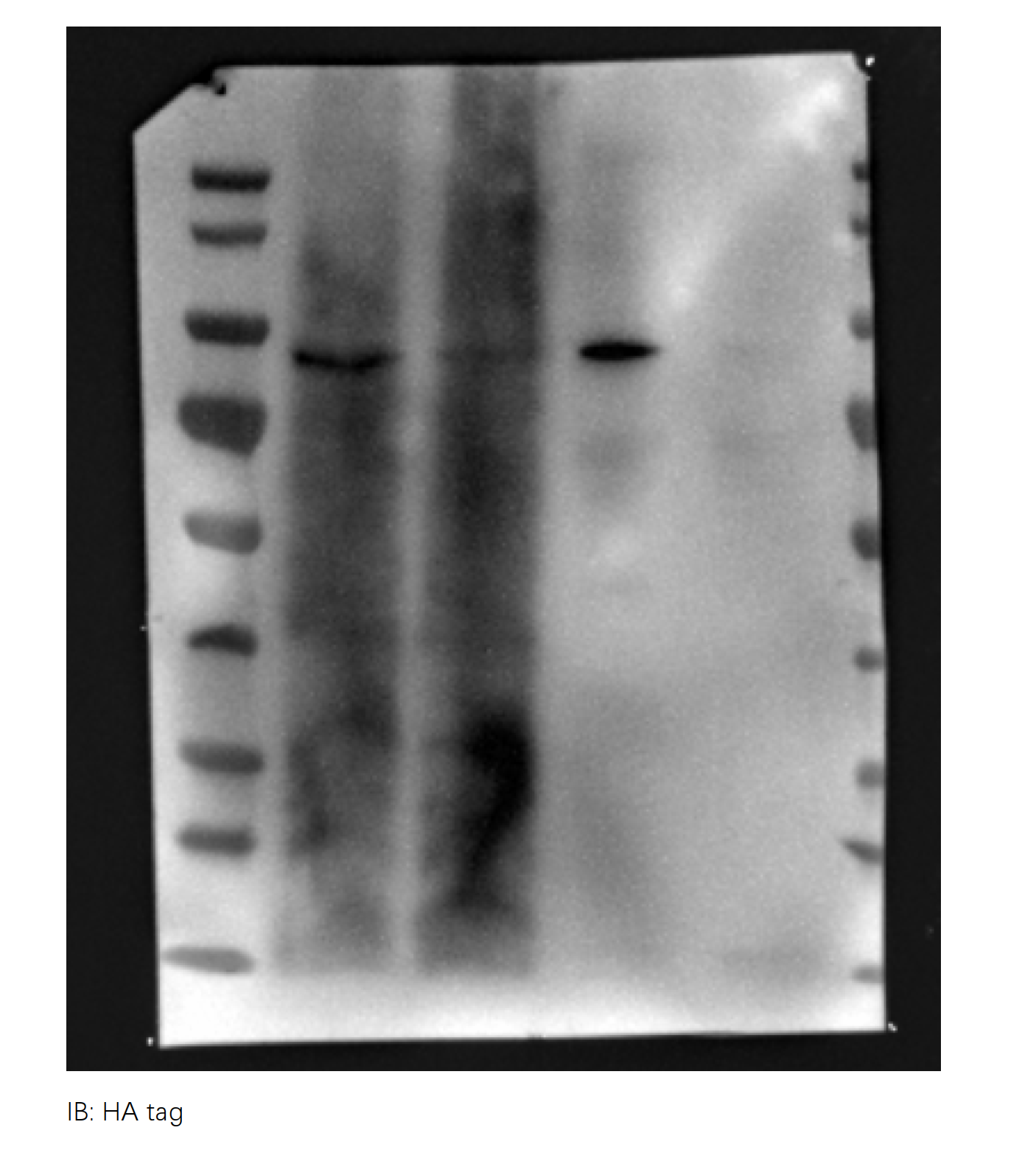

Supplement: Figure 1—figure supplement 1—source data 2. [file elife-98579-fig1-figsupp1-data2.zip › Figure 1-figure supplement 1-source data 2/IB HA.tif]

|      |   |     |   |           |
|------|---|-----|---|-----------|
| eVLP | + | +   | + | +         |
| DBCO | 0 | 3.5 | 7 | 14 (nmol) |
| CpG  | + | +   | + | +         |

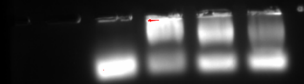

Supplement: Figure 2—source data 2. [file elife-98579-fig2-data2.pdf]

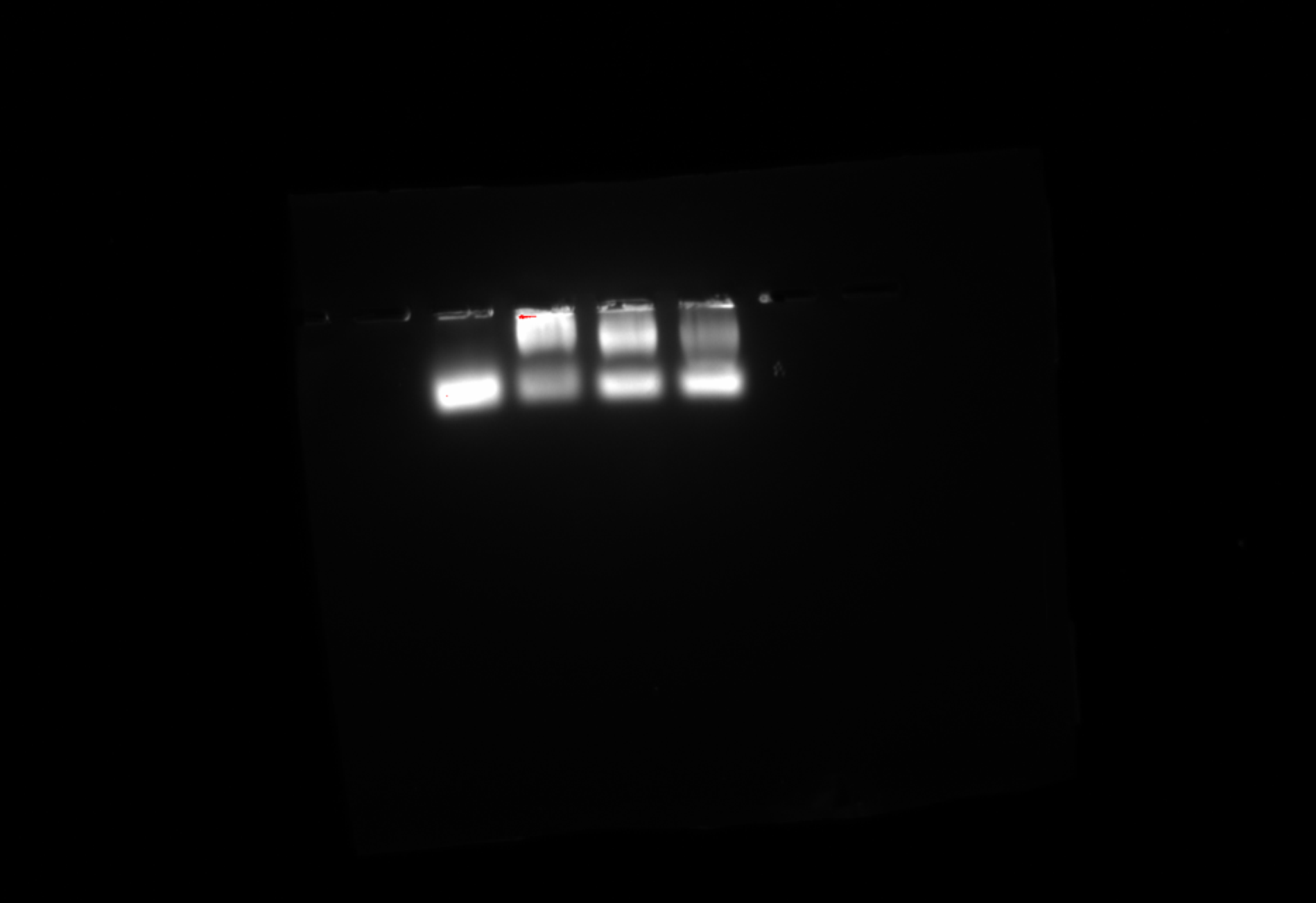

Supplement: Figure 2—source data 3. [file elife-98579-fig2-data3.tif]

|      |   |     |     |     |   |
|------|---|-----|-----|-----|---|
| eVLP | + | +   | +   | +   | + |
| DBCO | 0 | 0.7 | 2.1 | 3.5 | 7 |
| CpG  | + | +   | +   | +   | + |

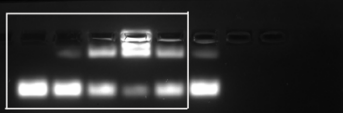

Supplement: Figure 2—figure supplement 2—source data 1. [file elife-98579-fig2-figsupp2-data1.pdf]

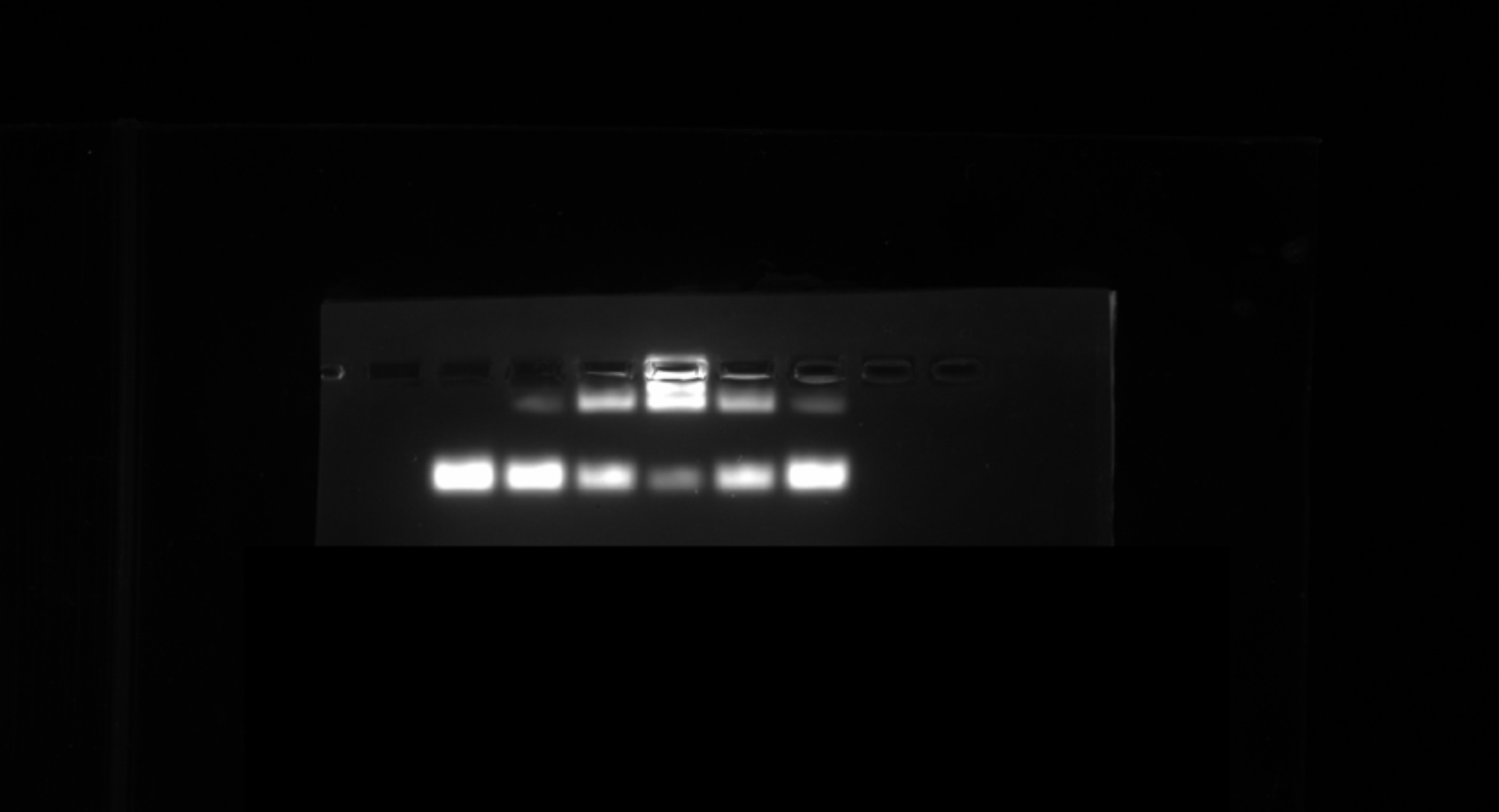

Supplement: Figure 2—figure supplement 2—source data 2. [file elife-98579-fig2-figsupp2-data2.tif]

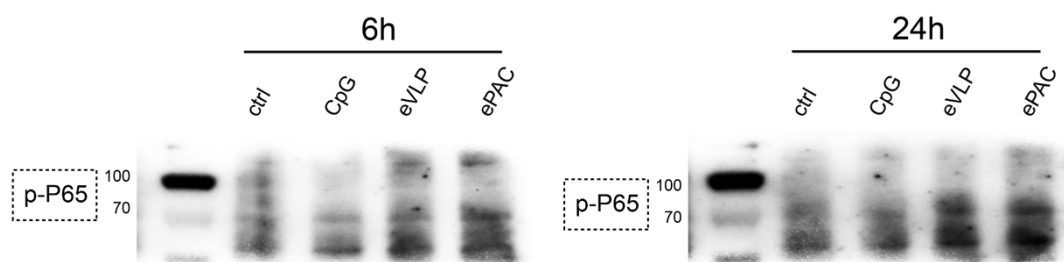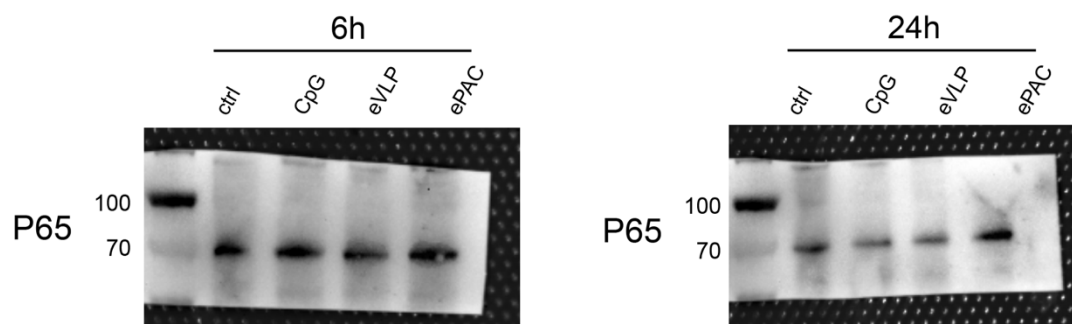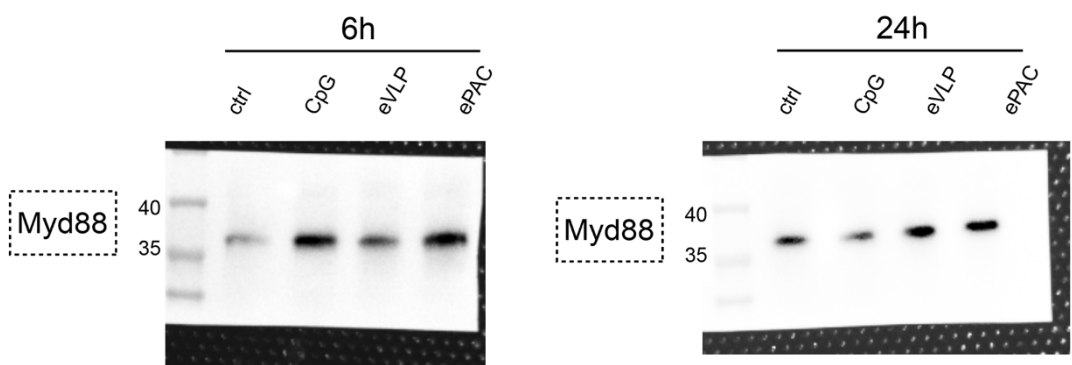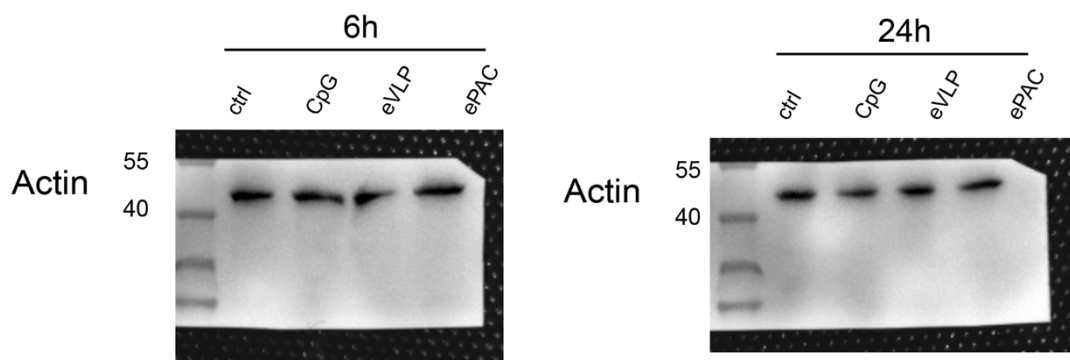

Supplement: Figure 3—source data 2. [file elife-98579-fig3-data2.pdf]

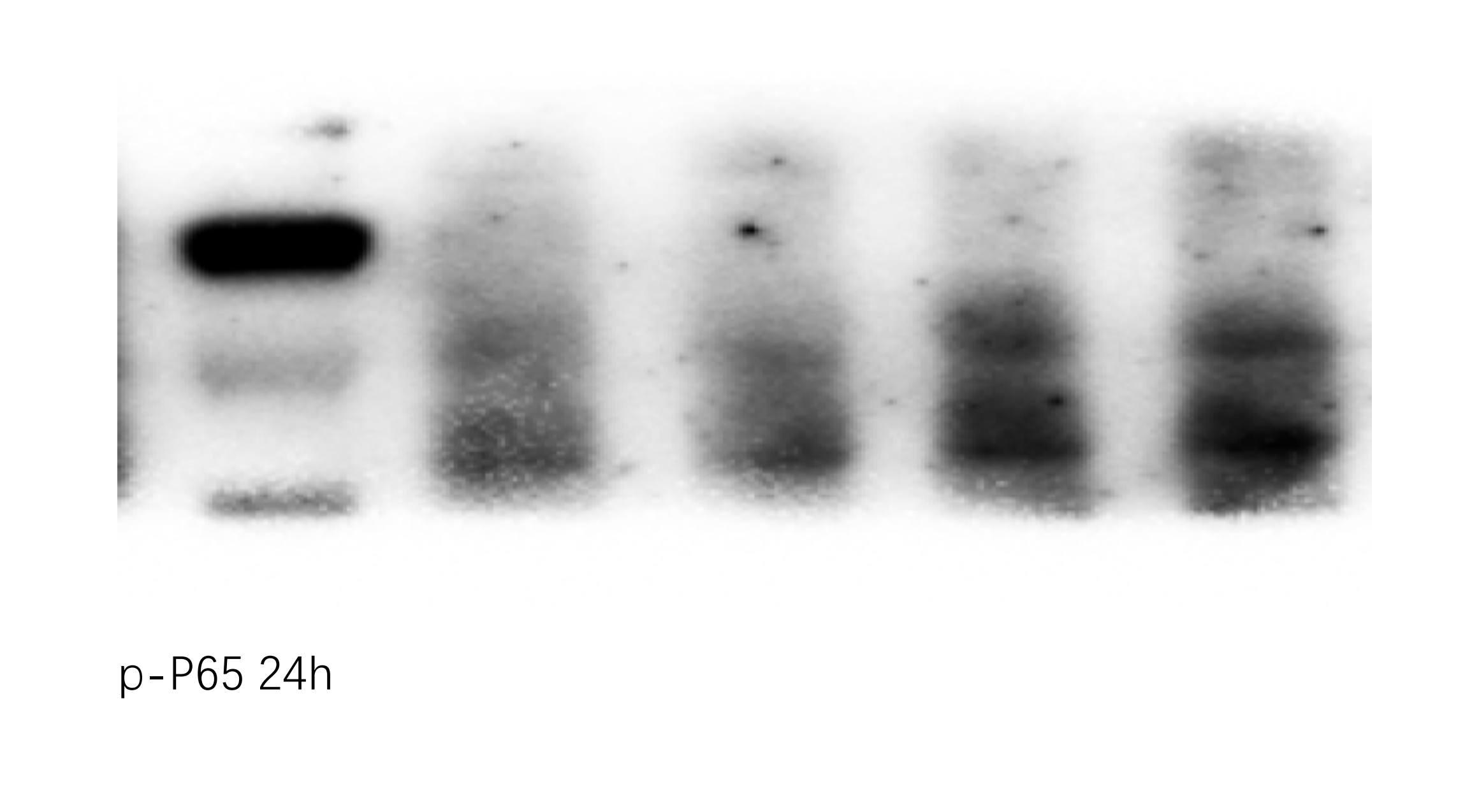

Supplement: Figure 3—source data 3. [file elife-98579-fig3-data3.zip › Figure 3-source data 3/p-P65 24h.tif]

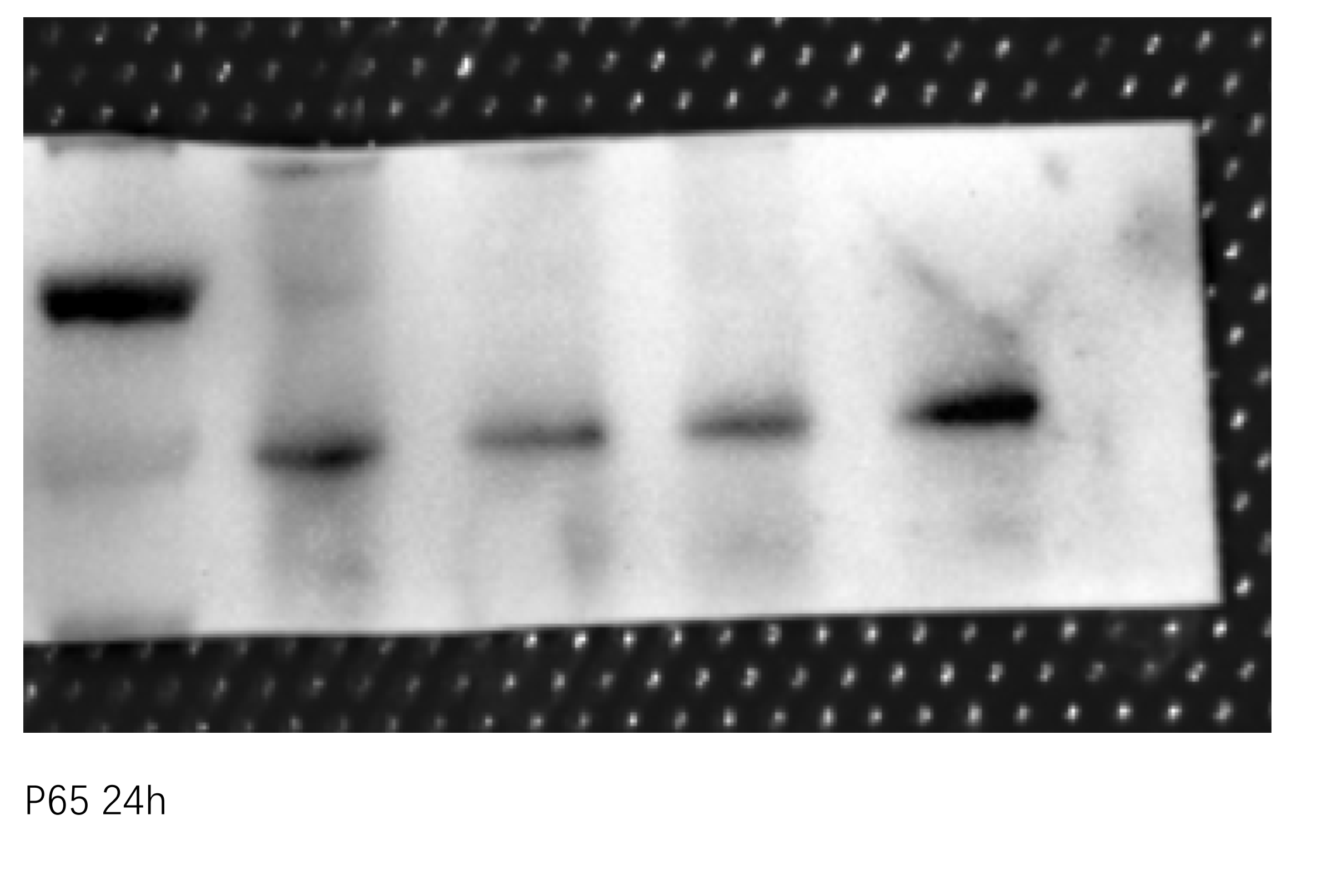

Supplement: Figure 3—source data 3. [file elife-98579-fig3-data3.zip › Figure 3-source data 3/P65 24h.tif]

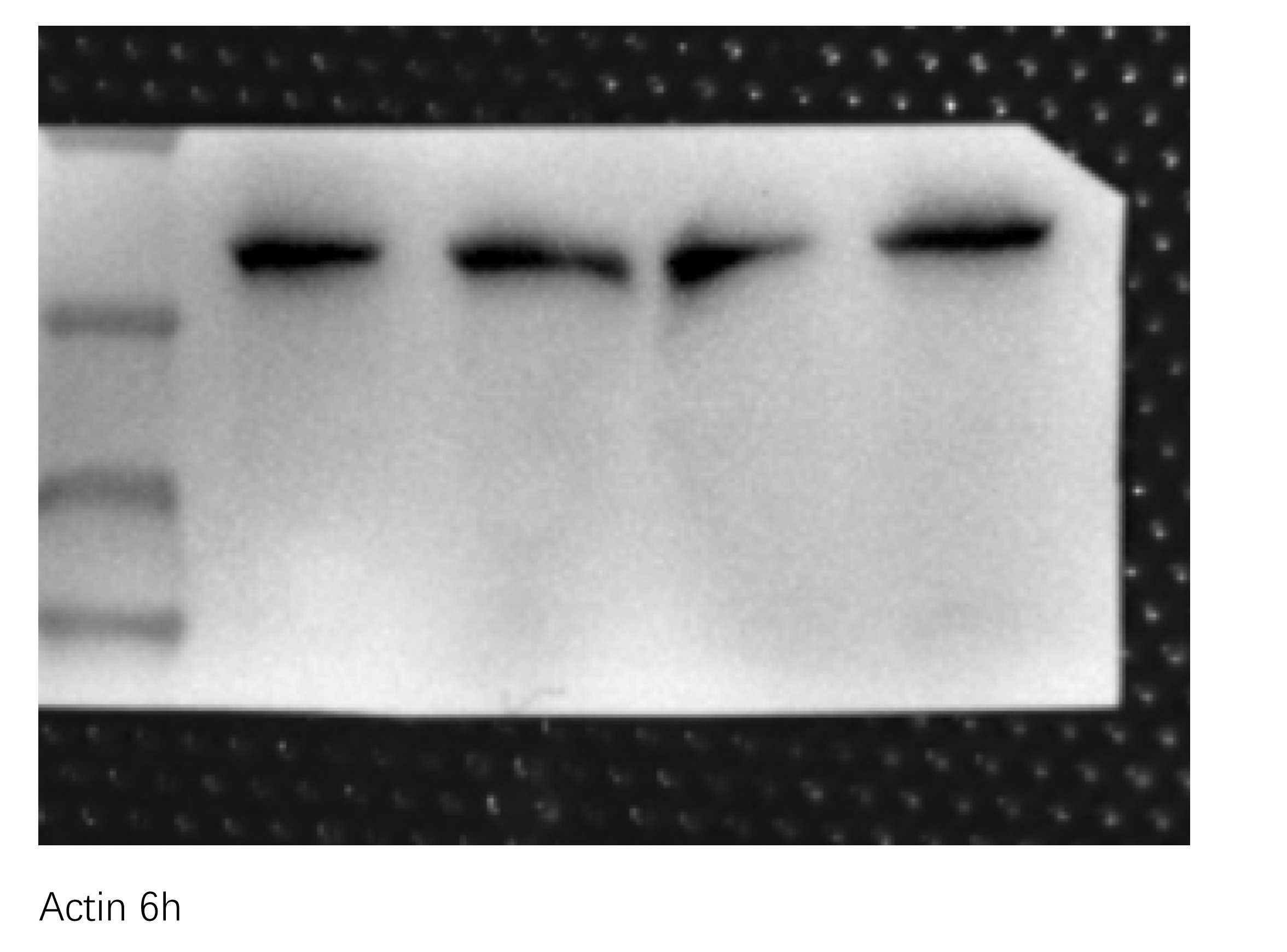

Supplement: Figure 3—source data 3. [file elife-98579-fig3-data3.zip › Figure 3-source data 3/Actin 5h.tif]

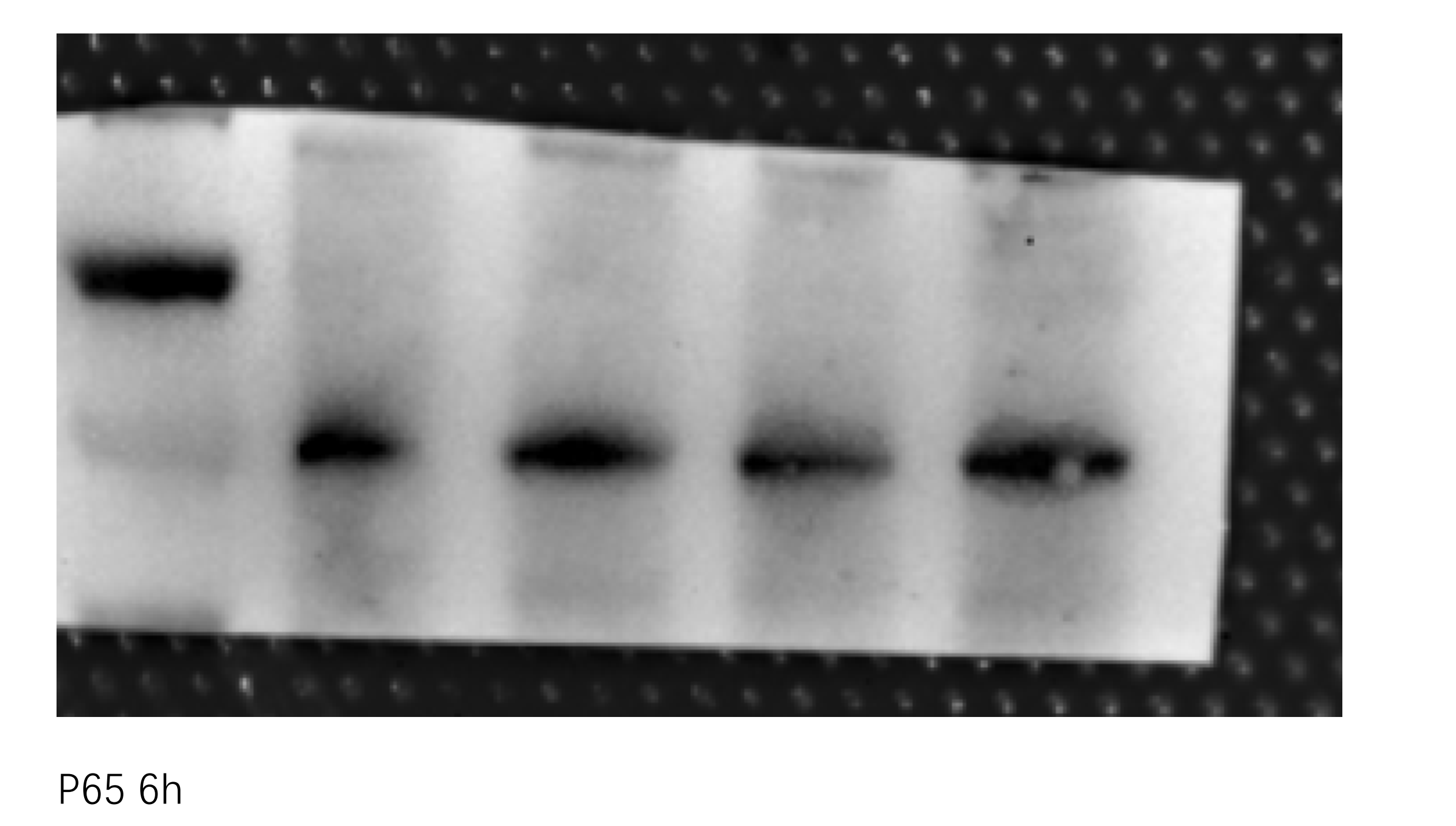

Supplement: Figure 3—source data 3. [file elife-98579-fig3-data3.zip › Figure 3-source data 3/P65 6h.tif]

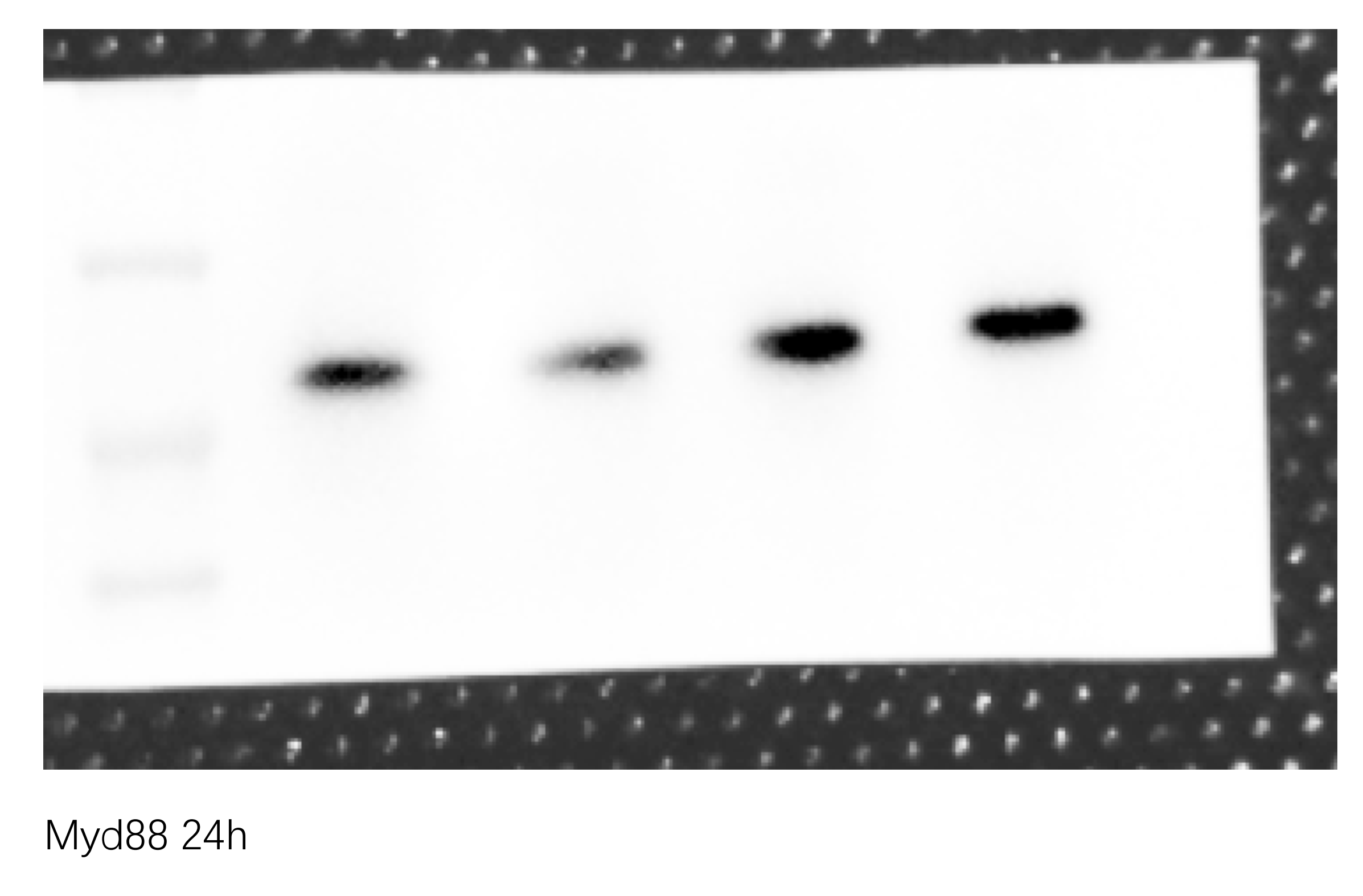

Supplement: Figure 3—source data 3. [file elife-98579-fig3-data3.zip › Figure 3-source data 3/Myd88 24h.tif]

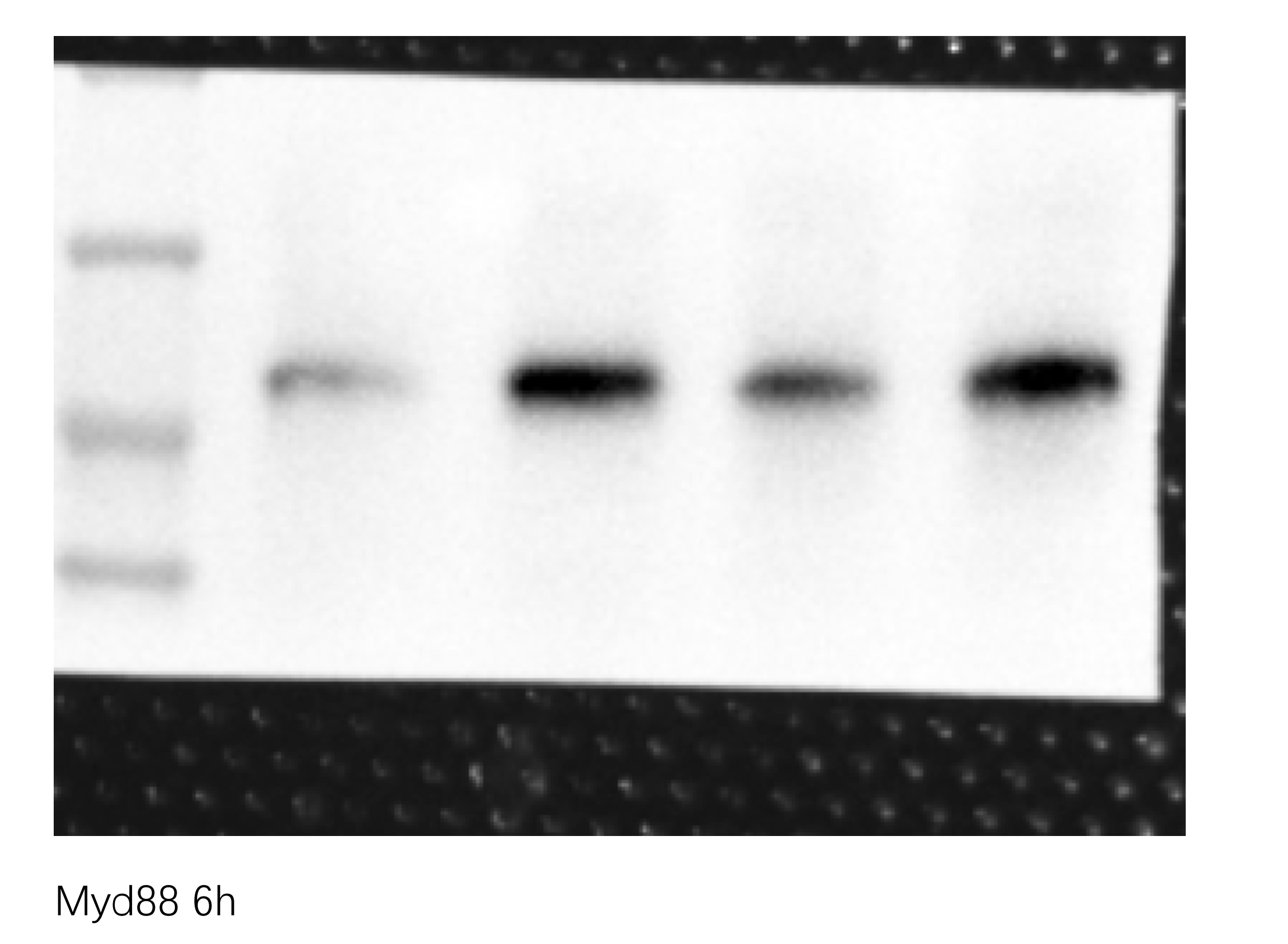

Supplement: Figure 3—source data 3. [file elife-98579-fig3-data3.zip › Figure 3-source data 3/Myd88 6h.tif]

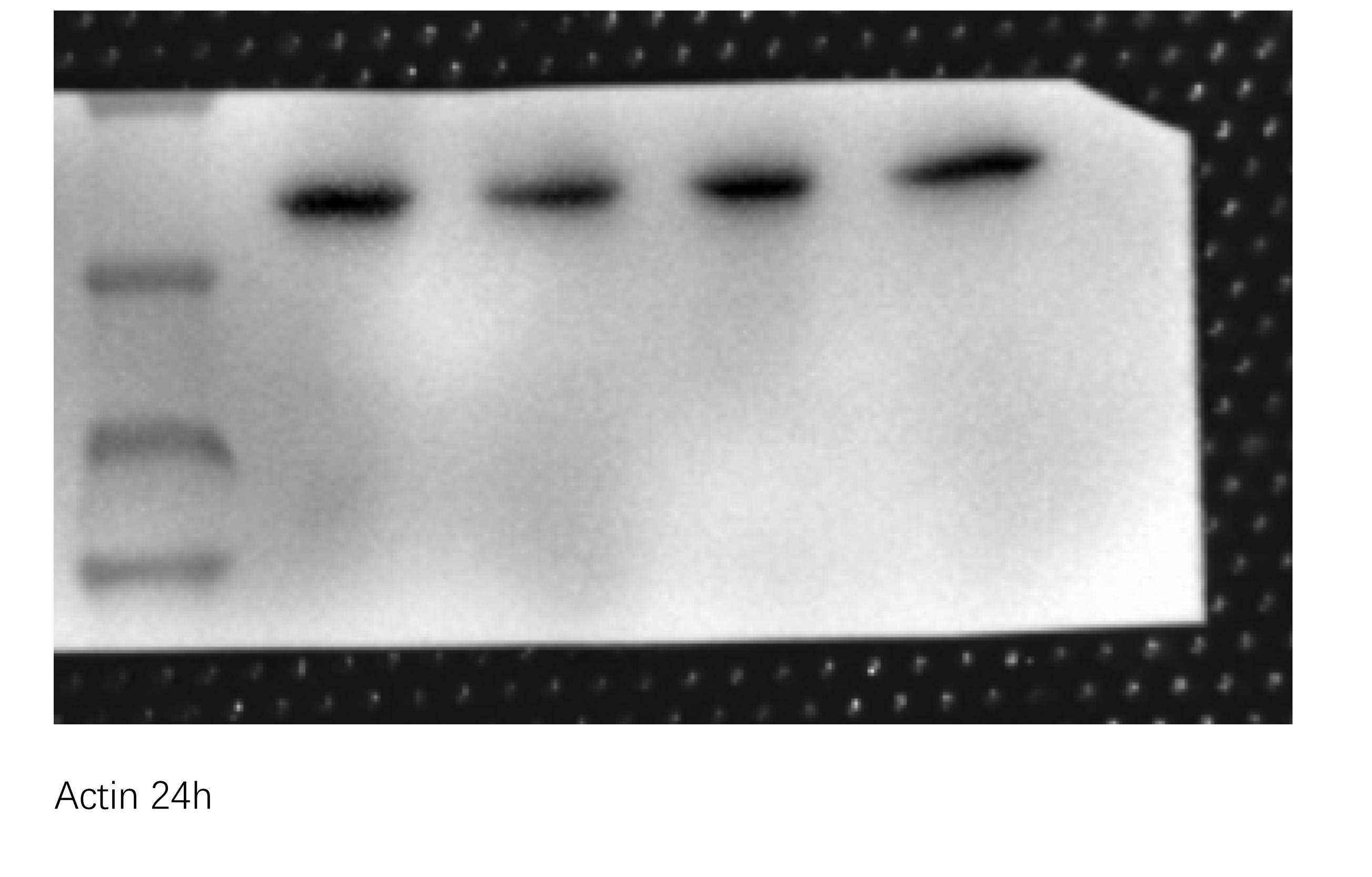

Supplement: Figure 3—source data 3. [file elife-98579-fig3-data3.zip › Figure 3-source data 3/Actin 24h.tif]

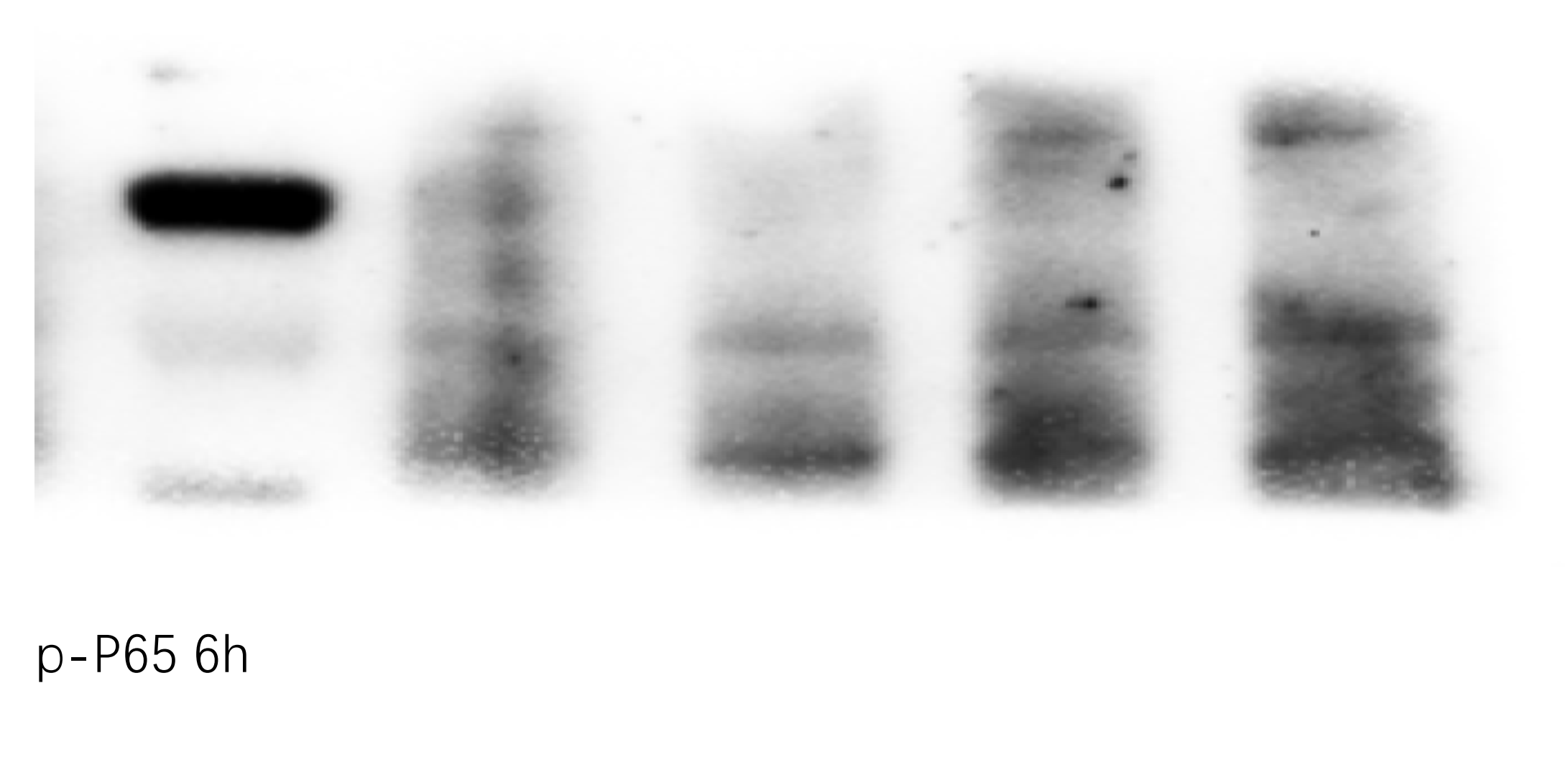

Supplement: Figure 3—source data 3. [file elife-98579-fig3-data3.zip › Figure 3-source data 3/p-P65 6h.tif]
