# Supplementary material for: Engineering PEG10-assembled endogenous virus-like particles with genetically encoded neoantigen peptides for cancer vaccination
Source: eLife. 2024 Sep 13;13:RP98579. doi: 10.7554/eLife.98579 (PMC11398863; doi:10.7554/eLife.98579)
Supplement: Figure 1—figure supplement 1—source data 1. [file elife-98579-fig1-figsupp1-data1.pdf]

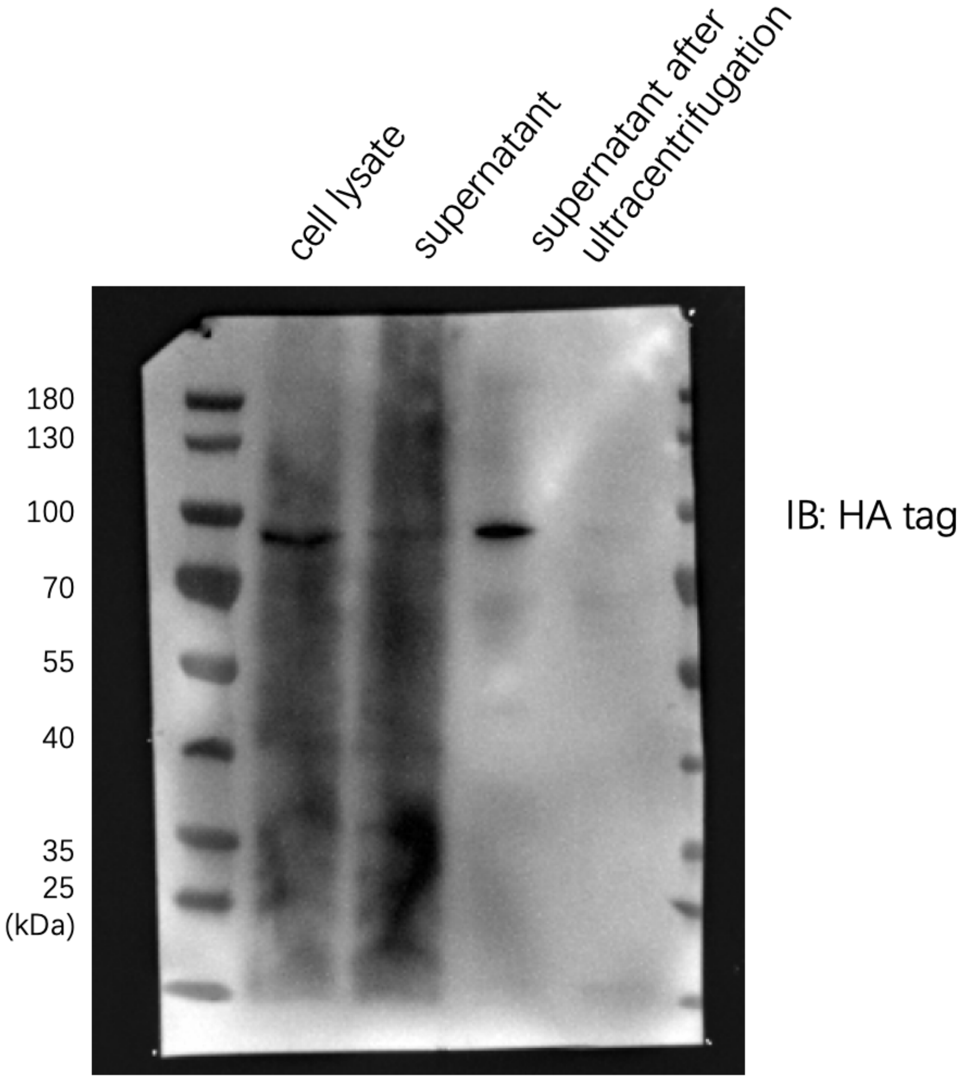

cell lysate
supernatant
supernatant after ultracentrifugation
180
130
100
70
55
40
35
25
(kDa)
IB: HA tag

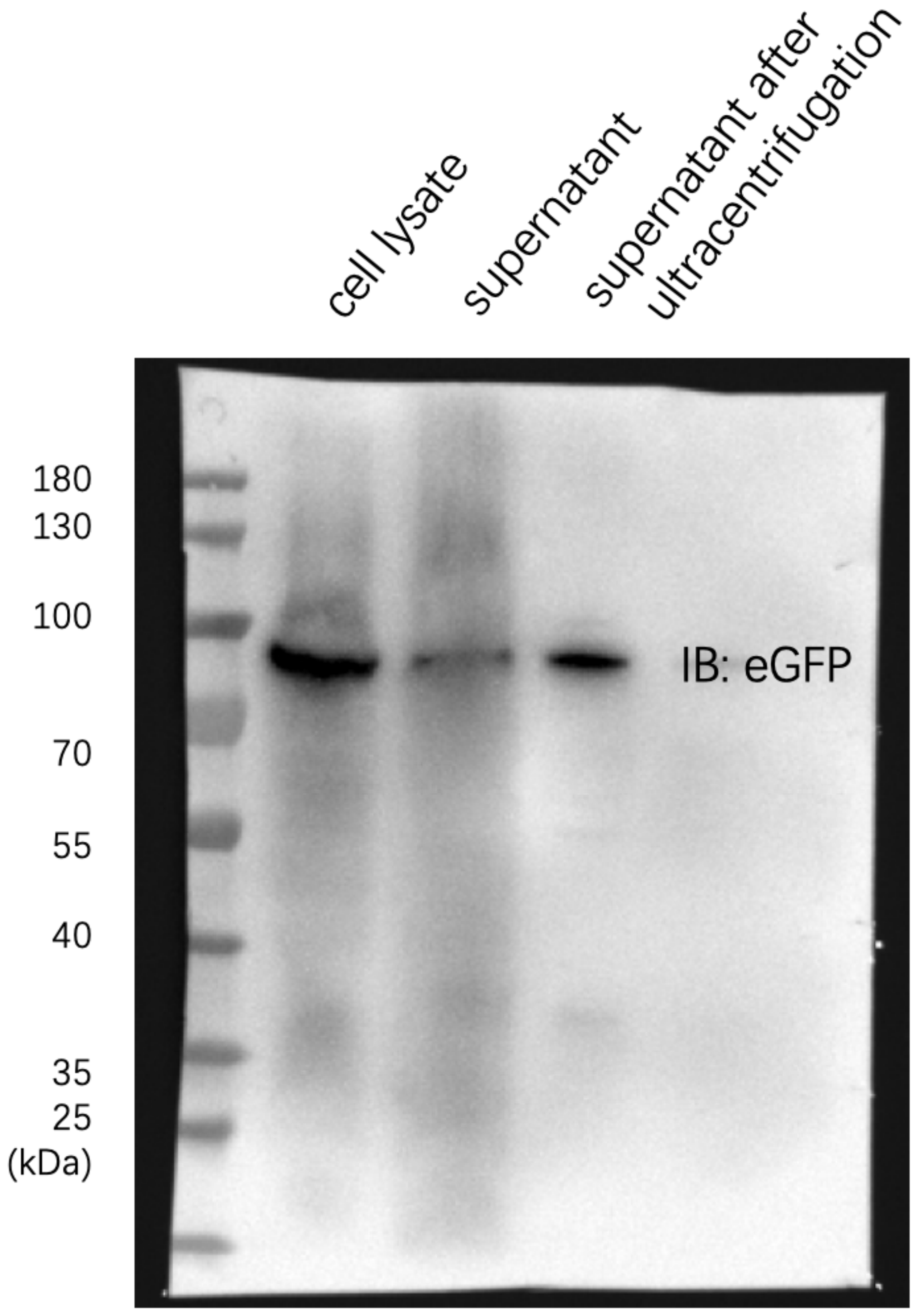
cell lysate
supernatant
supernatant after ultracentrifugation
180
130
100
70
55
40
35
25
(kDa)
IB: eGFP

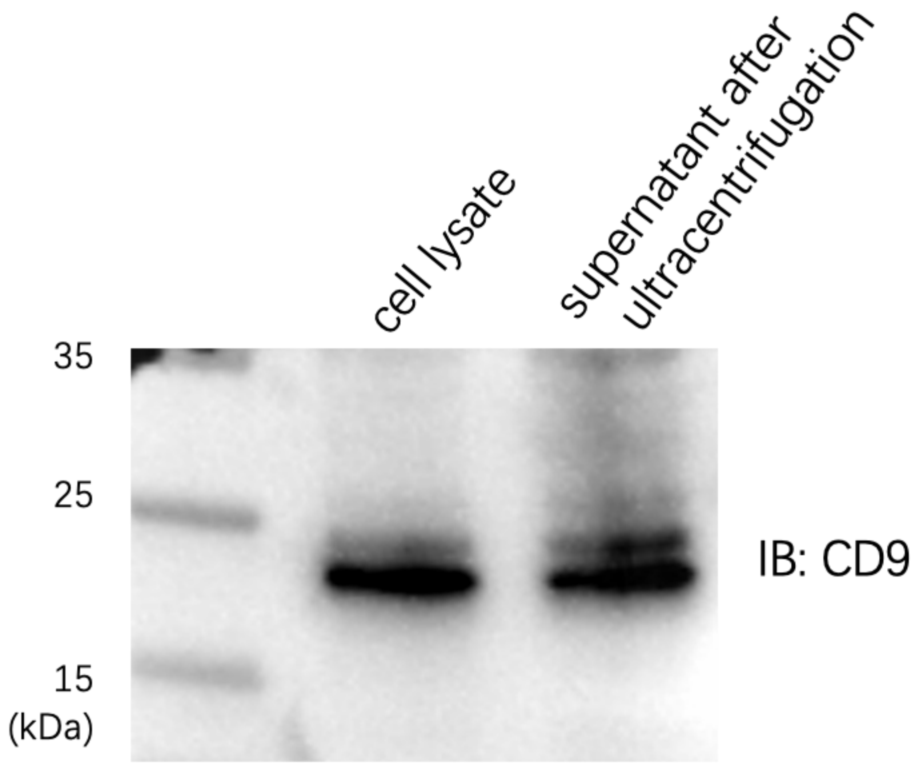
cell lysate
supernatant after ultracentrifugation
35
25
15
(kDa)
IB: CD9
