# Supplementary material for: Engineering PEG10-assembled endogenous virus-like particles with genetically encoded neoantigen peptides for cancer vaccination
Source: eLife. 2024 Sep 13;13:RP98579. doi: 10.7554/eLife.98579 (PMC11398863; doi:10.7554/eLife.98579)
Supplement: Supplementary file 1. — (a) The sequences of plasmids used in the study. (b) Antibodies used in the study. [file elife-98579-supp1.docx]

**Supplemental Information**

**Engineering PEG10 assembled endogenous** **virus-like particles with genetically encoded neoantigen peptides for cancer vaccination**

Ruijing Tang^1,2,3,5^, Luobin Guo^1,2,3,5^, Tingyu Wei^1,2,3^, Tingting Chen^1,2,3^, Huan Yang^1,2,3^, Honghao Ye^1,2,3^, Fangzhou Lin^1,2,3^, Yongyi Zeng^1,2,3^, Haijun Yu^4^, Zhixiong Cai^1,2,3,*^, and Xiaolong Liu^1,2,3,*^

1 The United Innovation of Mengchao Hepatobiliary Technology Key Laboratory of Fujian Province, Mengchao Hepatobiliary Hospital of Fujian Medical University, Fuzhou 350025, P. R. China;

2 The Liver Center of Fujian Province, Fujian Medical University, Fuzhou 350025, P. R. China;

3 Mengchao Med-X Center, Fuzhou University, Fuzhou 350025, P. R. China;

4 State Key Laboratory of Drug Research & Center of Pharmaceutics, Shanghai Institute of Materia Medica, Chinese Academy of Sciences, Shanghai 201203, P. R. China;

5 These authors contributed equally: Ruijing Tang, Luobin Guo.

*Correspondence: Xiaolong Liu (xiaoloong.liu@gmail.com, Mengchao Hepatobiliary Hospital of Fujian Medical University, Fuzhou 350025, P. R. China); Zhixiong Cai (caizhixiong1985@163.com, Mengchao Hepatobiliary Hospital of Fujian Medical University, Fuzhou 350025, P. R. China)

**Supplementary File 1a. The sequences of plasmids used in the study**

| 1. pcDNA3.1-gag-//-eGFP-HA: the sequence of gag-//-eGFP-HA as shown in right was inserted into the pCDH back bone through BglII and XhoI. | agatctgcattcgccaccatggctgctgcaggtggttcatcaaactgcccgccccctccccctccccctcctcccaacaacaacaacaacaacaacaccccaaagagcccaggcgtgcctgacgccgaagatgatgatgaacgcagacacgatgagctccctgaagacatcaacaactttgacgaagacatgaacaggcagtttgagaatatgaacctgctggatcaggtggagttgcttgcacagagctacagtctgctggatcatttagatgactttgatgatgatgatgaagacgatgactttgatccagaacctgaccaggatgagctccctgagtacagtgacgatgatgacctggagcttcagggtgctgcagcagcccctatcccaaactttttctccgatgatgactgccttgaagaccttcctgagaagttcgatggcaaccctgacatgctgggtcctttcatgtatcagtgccagctcttcatggaaaagagcaccagagatttctcagttgaccgcatccgtgtgtgcttcgtgacaagcatgctgatcggccgtgccgcccgctgggctactgccaagctgcaaagatgtacttacctgatgcacaactacactgcctttatgatggagctgaagcatgtctttgaagaccctcagagacgtgaagctgccaaacgcaagatcagacgtctgcgccagggccctgggcctgttgtggactactccaatgcattccagatgattgcccaggacctggattggactgagcctgccctgatggatcagttccaggaaggtctcaacccagacattcgcgcagagctgtctcgccaggaggcccccaagaccctggctgctctgattactgcctgtattcacatcgagagaaggctggctcgtgacgctgctgcaaagcccgatccttcacccagagccttggtgatgcctccaaacagccagaccgatcccaccgagcctgtgggaggtgcccgcatgcgcctgtccaaggaagaaaaggagagacgccgcaaaatgaatttgtgtctctactgtggcaatggaggccatttcgccgacacgtgtccagcgaaagcctccaagaattcgatggtgagcaagggcgaggagctgttcaccggggtggtgcccatcctggtcgagctggacggcgacgtaaacggccacaagttcagcgtgtccggcgagggcgagggcgatgccacctacggcaagctgaccctgaagttcatctgcaccaccggcaagctgcccgtgccctggcccaccctcgtgaccaccctgacctacggcgtgcagtgcttcagccgctaccccgaccacatgaagcagcacgacttcttcaagtccgccatgcccgaaggctacgtccaggagcgcaccatcttcttcaaggacgacggcaactacaagacccgcgccgaggtgaagttcgagggcgacaccctggtgaaccgcatcgagctgaagggcatcgacttcaaggaggacggcaacatcctggggcacaagctggagtacaactacaacagccacaacgtctatatcatggccgacaagcagaagaacggcatcaaggtgaacttcaagatccgccacaacatcgaggacggcagcgtgcagctcgccgaccactaccagcagaacacccccatcggcgacggccccgtgctgctgcccgacaaccactacctgagcacccagtccgccctgagcaaagaccccaacgagaagcgcgatcacatggtcctgctggagttcgtgaccgccgccgggatcactctcggcatggacgagctgtacaagtatccgtatgatgttccggattatgcatagtaactcgag |
| --- | --- |
| 1. pcDNA3.1-gag-GS-eGFP-HA: the sequence of gag-GS-eGFP-HA as shown in right was inserted into the pCDH back bone through BglII and XhoI. | agatctgcattcgccaccatggctgctgcaggtggttcatcaaactgcccgccccctccccctccccctcctcccaacaacaacaacaacaacaacaccccaaagagcccaggcgtgcctgacgccgaagatgatgatgaacgcagacacgatgagctccctgaagacatcaacaactttgacgaagacatgaacaggcagtttgagaatatgaacctgctggatcaggtggagttgcttgcacagagctacagtctgctggatcatttagatgactttgatgatgatgatgaagacgatgactttgatccagaacctgaccaggatgagctccctgagtacagtgacgatgatgacctggagcttcagggtgctgcagcagcccctatcccaaactttttctccgatgatgactgccttgaagaccttcctgagaagttcgatggcaaccctgacatgctgggtcctttcatgtatcagtgccagctcttcatggaaaagagcaccagagatttctcagttgaccgcatccgtgtgtgcttcgtgacaagcatgctgatcggccgtgccgcccgctgggctactgccaagctgcaaagatgtacttacctgatgcacaactacactgcctttatgatggagctgaagcatgtctttgaagaccctcagagacgtgaagctgccaaacgcaagatcagacgtctgcgccagggccctgggcctgttgtggactactccaatgcattccagatgattgcccaggacctggattggactgagcctgccctgatggatcagttccaggaaggtctcaacccagacattcgcgcagagctgtctcgccaggaggcccccaagaccctggctgctctgattactgcctgtattcacatcgagagaaggctggctcgtgacgctgctgcaaagcccgatccttcacccagagccttggtgatgcctccaaacaagaattcgggaggcggagggagcggaggcggagggagtggaggcggcggatctatggtgagcaagggcgaggagctgttcaccggggtggtgcccatcctggtcgagctggacggcgacgtaaacggccacaagttcagcgtgtccggcgagggcgagggcgatgccacctacggcaagctgaccctgaagttcatctgcaccaccggcaagctgcccgtgccctggcccaccctcgtgaccaccctgacctacggcgtgcagtgcttcagccgctaccccgaccacatgaagcagcacgacttcttcaagtccgccatgcccgaaggctacgtccaggagcgcaccatcttcttcaaggacgacggcaactacaagacccgcgccgaggtgaagttcgagggcgacaccctggtgaaccgcatcgagctgaagggcatcgacttcaaggaggacggcaacatcctggggcacaagctggagtacaactacaacagccacaacgtctatatcatggccgacaagcagaagaacggcatcaaggtgaacttcaagatccgccacaacatcgaggacggcagcgtgcagctcgccgaccactaccagcagaacacccccatcggcgacggccccgtgctgctgcccgacaaccactacctgagcacccagtccgccctgagcaaagaccccaacgagaagcgcgatcacatggtcctgctggagttcgtgaccgccgccgggatcactctcggcatggacgagctgtacaagtatccgtatgatgttccggattatgcatagtaactcgag |
| 1. pcDNA3.1-gag-//-pol-eGFP-HA: the sequence of gag-//-pol-eGFP-HA as shown in right was inserted into the pCDH back bone through BglII and XhaI. | agatctgcattcgccaccatggctgctgcaggtggttcatcaaactgcccgccccctccccctccccctcctcccaacaacaacaacaacaacaacaccccaaagagcccaggcgtgcctgacgccgaagatgatgatgaacgcagacacgatgagctccctgaagacatcaacaactttgacgaagacatgaacaggcagtttgagaatatgaacctgctggatcaggtggagttgcttgcacagagctacagtctgctggatcatttagatgactttgatgatgatgatgaagacgatgactttgatccagaacctgaccaggatgagctccctgagtacagtgacgatgatgacctggagcttcagggtgctgcagcagcccctatcccaaactttttctccgatgatgactgccttgaagaccttcctgagaagttcgatggcaaccctgacatgctgggtcctttcatgtatcagtgccagctcttcatggaaaagagcaccagagatttctcagttgaccgcatccgtgtgtgcttcgtgacaagcatgctgatcggccgtgccgcccgctgggctactgccaagctgcaaagatgtacttacctgatgcacaactacactgcctttatgatggagctgaagcatgtctttgaagaccctcagagacgtgaagctgccaaacgcaagatcagacgtctgcgccagggccctgggcctgttgtggactactccaatgcattccagatgattgcccaggacctggattggactgagcctgccctgatggatcagttccaggaaggtctcaacccagacattcgcgcagagctgtctcgccaggaggcccccaagaccctggctgctctgattactgcctgtattcacatcgagagaaggctggctcgtgacgctgctgcaaagcccgatccttcacccagagccttggtgatgcctccaaacagccagaccgatcccaccgagcctgtgggaggtgcccgcatgcgcctgtccaaggaagaaaaggagagacgccgcaaaatgaatttgtgtctctactgtggcaatggaggccatttcgccgacacgtgtccagcgaaagcctccaagaattcgccgccgggaaactccccggccccgctgtagggggaccttcagcgacagggccagaacgaataaggtccccaccctccgaggcttcgactcagcacctgcaagtgatgctccagattcatatgccgggcagacccaccctgtttgtccgagctatgattgattctggtgcatctggcaacttcattgatcaagactttgtcatacaaaatgcaattcctctcagaatcaaagactggccagtgatggtggaagctattgatgggcatccaattgcctcgggcccaatcattttggaaacccaccacctgatagttgatctgggagaccaccgtgagatactgtcatttgatgtgactcagtctccattctttcctattgtcctaggaatAcgttggctctctactcacgacccacacatcacctggagtactcgctccatagtcttcaactctgattactgtcggttgagatgccgaatgttcgctcagatcccaagcaacctgctctttaccgttccccaaccaaatctccacccctatctcctgcatcatgtacacccccatgtacaccctcacatgcatcaacaccttcatcaacacctgcatcagtttcttcatcctgatccacatcagtatccacacccagacccccattaccatcatcaccagcaggctgatatgcagcatcagctgcaacagtatttgtatcagtacttgtactaccatctgtaccctgtcatgcatcatcatcttcctcctgaccaacatgaacatctgcacgaatatcttcaccaatacctccatcagtaccttcaccaattcctccaccaccatcttcaccctgacttgcaccaatatttgtaccagtatcttcacaaccacatgaatccagatccacatcaccatccccatccagatccccctcaggatccacatcaccctccacatcaggatccccatcaggatcctccacatcaggatccacatcaggatgcacatcaggatccccatatggatccacacctgcatcagcaccagcatccgcagccgcagccgcatccacaacagcatcctaaccatcctcagcagccaccattcttctaccacatggctggattcagaatttaccaccctgtaaggtattactatattcagaatgtgtatacacctgttgatgagcatgtctatccgggtcaccgggtggttgaccctaacattgagatgattcctggagcgcacagcctgcccagtggacatttgtactcaatgtctgagtctgaaatgaatgctctgcgaaatttcgtggacaggaatgttaaagatgggctcatgactcccactgtggcgcccaatggagcccaagtcctgcaagtgaaaagagggtggaaactccaagtcacttacaattgccgagctccacagagtggcaccatccaaaatcagtacctacgcatgtctcttccaaatatgggagaccctgcacacctggcaagctatggtgaatttgtccaagttcctggctacccatatccagcctatgtttactatacaagcccgcatatgatgactgcgtggtacccagtaggacgagatgtacatggacgaataatcgttgtgcctgttgtaatcacctggtctcaaaatacgaaccgccagcctccggtgccccagtatcctcctccgcagccacctccaccaccaccaccacctccaccgccaccaccacctccaccagcatcatcctgcagtgctgcgggaggcggagggagcggaggcggagggagtggaggcggcggatctctcgaggccaccatggtgagcaagggcgaggagctgttcaccggggtggtgcccatcctggtcgagctggacggcgacgtaaacggccacaagttcagcgtgtccggcgagggcgagggcgatgccacctacggcaagctgaccctgaagttcatctgcaccaccggcaagctgcccgtgccctggcccaccctcgtgaccaccctgacctacggcgtgcagtgcttcagccgctaccccgaccacatgaagcagcacgacttcttcaagtccgccatgcccgaaggctacgtccaggagcgcaccatcttcttcaaggacgacggcaactacaagacccgcgccgaggtgaagttcgagggcgacaccctggtgaaccgcatcgagctgaagggcatcgacttcaaggaggacggcaacatcctggggcacaagctggagtacaactacaacagccacaacgtctatatcatggccgacaagcagaagaacggcatcaaggtgaacttcaagatccgccacaacatcgaggacggcagcgtgcagctcgccgaccactaccagcagaacacccccatcggcgacggccccgtgctgctgcccgacaaccactacctgagcacccagtccgccctgagcaaagaccccaacgagaagcgcgatcacatggtcctgctggagttcgtgaccgccgccgggatcactctcggcatggacgagctgtacaagtatccgtatgatgttccggattatgcatagtaatctaga |
| 1. pcDNA3.1-gag-neoantigens: the sequence of gag-neoantigens as shown in right was inserted into the pCDH back bone through BglII and XhoI. | agatctgcattcgccaccatggctgctgcaggtggttcatcaaactgcccgccccctccccctccccctcctcccaacaacaacaacaacaacaacaccccaaagagcccaggcgtgcctgacgccgaagatgatgatgaacgcagacacgatgagctccctgaagacatcaacaactttgacgaagacatgaacaggcagtttgagaatatgaacctgctggatcaggtggagttgcttgcacagagctacagtctgctggatcatttagatgactttgatgatgatgatgaagacgatgactttgatccagaacctgaccaggatgagctccctgagtacagtgacgatgatgacctggagcttcagggtgctgcagcagcccctatcccaaactttttctccgatgatgactgccttgaagaccttcctgagaagttcgatggcaaccctgacatgctgggtcctttcatgtatcagtgccagctcttcatggaaaagagcaccagagatttctcagttgaccgcatccgtgtgtgcttcgtgacaagcatgctgatcggccgtgccgcccgctgggctactgccaagctgcaaagatgtacttacctgatgcacaactacactgcctttatgatggagctgaagcatgtctttgaagaccctcagagacgtgaagctgccaaacgcaagatcagacgtctgcgccagggccctgggcctgttgtggactactccaatgcattccagatgattgcccaggacctggattggactgagcctgccctgatggatcagttccaggaaggtctcaacccagacattcgcgcagagctgtctcgccaggaggcccccaagaccctggctgctctgattactgcctgtattcacatcgagagaaggctggctcgtgacgctgctgcaaagcccgatccttcacccagagccttggtgatgcctccaaacagccagaccgatcccaccgagcctgtgggaggtgcccgcatgcgcctgtccaaggaagaaaaggagagacgccgcaaaatgaatttgtgtctctactgtggcaatggaggccatttcgccgacacgtgtccagcgaaagcctccaagaattcgggaggcggagggagcggaggcggagggagtggaggcggcggatctatgaaggcccgaaactacctgcagtttctgccctcgaaaaccaaggtggctggaggcggagggagccgaggagaacactaccggtacaaggtcagcctccccgggggccagcacgccggaggcggagggagccatgttctctgggacttaaagcagatgtttcggtgtgctgtcttgaaaaacggaggcggagggagctgggacacttgtaccacttacaagtggcaaaagacactggaaggtcatgatggaggcggagggagcctatcaacgtacagaacagcttgcacgttacgatttgtacagaagcgatgcggaggcggagggagcctgtacactcacttcctgcagttgccactggcagccaccgggttctccgtgggaggcggagggagcaaaaggtggttatattggcaacctactctcactaagatggggtttgtgtcatgataactcgag |
| 1. pCDH-DEC-205-mCherry: the sequence of DEC-205-mCherry as shown in right was inserted into the pCDH back bone through XbaI and NotI. | tctagagccaccatgcggacgggccgggtgaccccgggcctggcggcggggctactcctgctgttgctgcggtccttcgggcttgtggagccttctgagagctcaggtaatgatccattcaccatcgtccatgaaaacactggcaagtgcatccagccgctgtctgactgggtagtggcccaggactgtagcggaactaacaacatgttgtggaagtgggtgtcccagcaccgcctctttcacctggaatcccagaagtgcctcggcctcgatattaccaaagccacggacaacctgcgaatgttcagctgtgactccaccgtcatgctgtggtggaaatgtgagcaccattcgctgtacaccgctgcccagtacaggctagctctgaaagatggatatgccgtagccaatacgaatacatctgatgtctggaagaagggaggctccgaggaaaacctttgtgcccagccttatcatgagatatacaccagagatgggaattcctacgggagaccttgtgaattccctttcttgattggtgagacatggtaccatgactgcattcatgatgaagatcatagtgggccatggtgtgccactaccctaagttatgaatatgatcaaaagtggggcatctgcctactaccagaaagtggctgtgaaggtaactgggaaaagaatgagcagattggaagttgctaccaatttaataatcaggaaattctgtcttggaaagaagcttatgtttcctgtcagaaccaaggagctgacttactgagcatccacagtgctgccgaattagcctacattacgggaaaagaggacattgctagacttgtttggcttggactgaatcagctctattctgcgagaggttgggaatggtcagacttcaggccactcaaatttcttaactgggatccaggcacgcccgttgcacctgtgattggtgggtcaagctgtgccagaatggacacagagtccgggctgtggcaaagtgtttcctgtgaatctcagcagccttacgtctgcaagaagccactgaacaacacgctggagctcccagatgtttggacttacacagatacccactgccatgtgggctggctgccaaataatgggttttgctatctgctggcgaatgaaagtagttcctgggatgcagcacatttgaaatgcaaagccttcggtgcagacctcatcagcatgcactccttagcagatgtggaggtggttgtcacgaaactccataatggggatgtcaaaaaagaaatatggacaggccttaaaaacacaaacagccctgctttgttccagtggtcggacggaacggaagttactctaacgtactggaatgagaatgagccgagtgttcccttcaacaagactcccaactgtgtttcctatttaggaaagttaggtcagtggaaagtccagtcctgtgagaagaaactcagatatgtatgcaagaaaaagggagaaataactaaggatgcagagtcggataagctgtgtccgccagacgagggctggaagagacatggagaaacctgttacaagatttatgagaaagaggcccctttcggaacgaactgcaacctgaccatcactagcaggttcgagcaggaattcttgaattatatgatgaagaactatgataagtcccttcggaagtacttctggactggcctgagagaccctgactctcgaggagaatacagttgggccgttgctcagggagtaaagcaggctgtgaccttttccaactggaattttcttgaaccggcgtctccaggcgggtgcgtggctatgtctactggaaagactcttggcaagtgggaagtgaagaactgcagaagcttccgtgctctttcaatatgcaagaaagtgagcgaaccccaggagcctgaagaagcagcccccaagcccgacgacccctgtcctgaaggctggcacactttcccctccagcctttcttgttataaggtgttccatatagaaagaatcgtaagaaagaggaactgggaagaagccgaaaggttctgccaagcccttggagctcacctacccagcttcagtcgtagagaggaaattaaggactttgtgcatttgttaaaggaccagttcagtgggcagcgttggttgtggattggtctgaataagagaagccctgatttacaagggtcctggcagtggagtgaccggacaccagtgtctgctgtgatgatggagccggagtttcaacaggattttgacatcagagactgtgctgccatcaaggtccttgatgtaccttggcgaagagtctggcatctctatgaggacaaggactatgcttactggaaaccttttgcttgtgatgccaagcttgagtgggtgtgccagattccaaaaggtagcactccccagatgccagactggtataatccagagcgcactggaattcatgggcccccagttataattgaaggaagtgaatactggtttgttgctgatccccacttaaactacgaagaagccgtcttatactgtgctagcaatcacagctttcttgccacgataacatcgttcacaggactaaaagctatcaaaaacaaactagcaaatatttctggcgaggaacagaagtggtgggtgaaaacgagtgagaatccaattgatcgttactttctaggctcgcgccgccgcctgtggcaccatttccccatgacgtttggagatgaatgtttgcacatgtcagccaagacgtggcttgttgacttaagtaaacgagcggactgtaatgccaagttgcccttcatctgtgaaagatacaatgtctcttcattagagaaatacagcccagatcctgcagccaaagtacagtgcactgagaagtggattccttttcaaaataagtgcttcctaaaggtcaactctgggcccgttacgttttctcaagcaagcggcatttgtcattcctacggcggcacccttccttccgtgctgagccggggtgaacaagatttcattatatccttgcttcctgaaatggaagctagtctatggattggtctgcgctggactgcctacgaaaggataaacagatggacagacaacagagagctgacctacagcaactttcacccactgctggtcggtcggaggctgagcataccaacgaatttctttgatgatgagtcccacttccactgcgccttgattcttaatctcaaaaagtcaccgcttactgggacctggaattttacttcctgttcagaacgacactctctgtctctctgtcaaaaatactcagagactgaagacggacagccctgggagaacacttcaaaaacagtgaagtatctaaataacctatacaaaatcatctcgaagcccctgacgtggcacggcgctctgaaggagtgcatgaaagagaagatgaggttggtgagcatcacagacccttaccagcaggccttcctcgcagtgcaggccaccctgcgcaacagctccttctggatcggactctccagtcaagatgatgaactcaactttggttggtcagatgggaaacgtcttcaatttagtaactgggctggaagcaatgagcaacttgatgactgcgtgatattagacacagatggattctggaaaacagctgactgtgatgataaccagcctggcgccatttgctactatccaggaaatgagactgaggaggaggtcagagcactggacactgctaaatgcccgtctcctgtacagagcaccccatggataccattccagaactcctgctacaatttcatgattaccaacaacaggcataagacagtcacaccggaggaagtgcagtccacgtgcgagaagctgcattcgaaagcacacagtctgagcattcggaatgaggaggagaatacctttgttgtggaacagcttctgtacttcaattatattgcctcatgggtcatgttaggaataacctatgaaaacaattctttgatgtggtttgataaaactgcattgtcctacacacactggagaacgggaagaccaactgtgaaaaatggcaaatttttggctggtctaagtactgatggattctgggatattcagtctttcaatgttattgaagaaacacttcatttttaccagcacagtatttctgcttgtaaaattgaaatggttgactatgaggacaaacacaatggcaccctgccacagttcattccatataaggacggcgtctacagcgttattcagaagaaggtgacgtggtatgaagcattgaacgcgtgctctcaaagtgggggagagttggccagtgttcacaacccaaatgggaagctctttctggaagacattgtgaaccgtgacggattccctctctgggttgggctctcaagtcatgatggaagcgaatcgagtttcgaatggtccgatggcagagcatttgactatgtcccatggcagagcctacaatctcccggagactgtgtcgtcttatatccaaaaggaatttggagacgtgaaaaatgcctgtctgttaaggatggtgctatttgttacaagcctacaaaagataaaaagctgatctttcatgtaaaatcatcaaaatgtccagtggcaaagagggatggtccccagtgggtccagtatgggggccactgttacgcttcggaccaggtactgcacagcttctcagaggccaaacaagtgtgtcaagagcttgatcattcggcaactgttgtcaccatagcagatgaaaatgagaataagtttgtgagcagactgatgagggagaactataatattactatgagagtttggcttggcctgtctcagcattcactcgatcagtcttggagttggctcgatggattagatgtgacatttgtcaaatgggaaaataaaactaaggatggtgatgggaaatgtagcattttaatagcttcaaatgaaacctggagaaaagtccattgctcacgtggctatgcaagagctgtctgcaaaattcctctgagcccggactacacaggcatagccatcctgtttgccgtgctgtgcctcttagggctcatcagcttggcgatttggttcctcttgcaacgatcccatatccgctggaccggcttctcctcggttcggtatgaacatggaaccaacgaagacgaggtgatgctcccttctttccacgacaccggtggttctggaggttcaatggtgagcaagggcgaggaggataacatggccatcatcaaggagttcatgcgcttcaaggtgcacatggagggctccgtgaacggccacgagttcgagatcgagggcgagggcgagggccgcccctacgagggcacccagaccgccaagctgaaggtgaccaagggtggccccctgcccttcgcctgggacatcctgtcccctcagttcatgtacggctccaaggcctacgtgaagcaccccgccgacatccccgactacttgaagctgtccttccccgagggcttcaagtgggagcgcgtgatgaacttcgaggacggcggcgtggtgaccgtgacccaggactcctccctgcaggacggcgagttcatctacaaggtgaagctgcgcggcaccaacttcccctccgacggccccgtaatgcagaagaagaccatgggctgggaggcctcctccgagcggatgtaccccgaggacggcgccctgaagggcgagatcaagcagaggctgaagctgaaggacggcggccactacgacgctgaggtcaagaccacctacaaggccaagaagcccgtgcagctgcccggcgcctacaacgtcaacatcaagttggacatcacctcccacaacgaggactacaccatcgtggaacagtacgaacgcgccgagggccgccactccaccggcggcatggacgagctgtacaagtaatgagcggccgc |
| 1. pcDNA3.1-gag-HBc 18-27: the sequence of gag-HBc 18-27 as shown in right was inserted into the pCDH back bone through BglII and XhoI. | agatctgcattcgccaccatggctgctgcaggtggttcatcaaactgcccgccccctccccctccccctcctcccaacaacaacaacaacaacaacaccccaaagagcccaggcgtgcctgacgccgaagatgatgatgaacgcagacacgatgagctccctgaagacatcaacaactttgacgaagacatgaacaggcagtttgagaatatgaacctgctggatcaggtggagttgcttgcacagagctacagtctgctggatcatttagatgactttgatgatgatgatgaagacgatgactttgatccagaacctgaccaggatgagctccctgagtacagtgacgatgatgacctggagcttcagggtgctgcagcagcccctatcccaaactttttctccgatgatgactgccttgaagaccttcctgagaagttcgatggcaaccctgacatgctgggtcctttcatgtatcagtgccagctcttcatggaaaagagcaccagagatttctcagttgaccgcatccgtgtgtgcttcgtgacaagcatgctgatcggccgtgccgcccgctgggctactgccaagctgcaaagatgtacttacctgatgcacaactacactgcctttatgatggagctgaagcatgtctttgaagaccctcagagacgtgaagctgccaaacgcaagatcagacgtctgcgccagggccctgggcctgttgtggactactccaatgcattccagatgattgcccaggacctggattggactgagcctgccctgatggatcagttccaggaaggtctcaacccagacattcgcgcagagctgtctcgccaggaggcccccaagaccctggctgctctgattactgcctgtattcacatcgagagaaggctggctcgtgacgctgctgcaaagcccgatccttcacccagagccttggtgatgcctccaaacagccagaccgatcccaccgagcctgtgggaggtgcccgcatgcgcctgtccaaggaagaaaaggagagacgccgcaaaatgaatttgtgtctctactgtggcaatggaggccatttcgccgacacgtgtccagcgaaagcctccaagaattcgggaggcggagggagctttcttccaagcgacttctttccaagtgtgtgataactcgag |

**Supplementary File 1b. Antibodies used in the study**

| Target | Clone | Source | Usage |
| --- | --- | --- | --- |
| CD11c-APC | N418 | Biolegend (#117310) | Flow cytometry |
| CD80-PE | 16-10A1 | Biolegend (#104708) | Flow cytometry |
| CD86-PeCy7 | GL-1 | Biolegend (#105014) | Flow cytometry |
| MHC-II-PE | M5/114.15.2 | eBioscience (#17-5321-82) | Flow cytometry |
| CD3-FITC | 17A2 | Biolegend (#100204) | Flow cytometry |
| CD8-APC | 53-5.8 | Biolegend (#140410) | Flow cytometry |
| CD44-PeCy7 | IM7 | Biolegend (#103030) | Flow cytometry |
| CD62L-PerCP | MEL-14 | Biolegend (#104430) | Flow cytometry |
| 4-1BB-APC | 17B5 | Biolegend (#106110) | Flow cytometry |
| 4-1BB-APC | 4B4-1 | BD Pharmingen (#550890) | Flow cytometry |
| HLA-A*02-FITC | BB7.2 | Abcam (#ab27728) | Flow cytometry |
| CD4 | EPR19514 | Abcam (#ab183685) | Immunohistochemistry |
| CD8 | EPR21769 | Abcam (#ab217344) | Immunohistochemistry |
| CD8 | EPR21769 | Abcam (#ab217344) | Immunofluorescence |
| PD-1 | EPR20665 | Abcam (#ab214421) | Immunofluorescence |
| TIM-3 | EPR22241 | Abcam (#ab241332) | Immunofluorescence |
| CTLA-4 | CAL49 | Abcam (#ab237712) | Immunofluorescence |
| HA tag | C29F4 | CST (#3724) | Western Blot |
| GFP | D5.1 | CST (#2956) | Western Blot |
| P65 | D14E12 | CST (#8242) | Western Blot |
| phospho-P65 | 93H1 | CST (#3033) | Western Blot |
| Myd88 | D80F5 | CST (#4283) | Western Blot |
| Actin | 13E5 | CST (#4970) | Western Blot |
| CD9 | E8L5J | CST (#98327) | Western Blot |
