# Supplementary material for: Engineering PEG10-assembled endogenous virus-like particles with genetically encoded neoantigen peptides for cancer vaccination
Source: eLife. 2024 Sep 13;13:RP98579. doi: 10.7554/eLife.98579 (PMC11398863; doi:10.7554/eLife.98579)
Supplement: Peer review history

# Editor report: eLife assessment

**DOI:** 10.7554/eLife.98579.3.sa0

**Authors:** Yunlu Dai

**Affiliations:** University of Macau Macao

**Keywords:** Convincing, Valuable

This study presents a **valuable** strategy to co-deliver peptides and adjuvants to antigen-presenting cells by engineering the Virus-like particle (VLP). The evidence supporting the claims of the authors is **convincing**, but the antitumour efficacy is unimpressive and would benefit from more antitumor experiments. The work will be of broad interest to bioengineers and medical biologists focusing on cancer vaccines.

# Referee report: Reviewer #1 (Public review)

**DOI:** 10.7554/eLife.98579.3.sa1

Summary:

The authors fabricated a novel cancer vaccine using endogenous virus-like particles with tumor neoantigen. The vaccine ePAC was proven to elicit strong immune stimulation with an increased killing effect against tumor cells in 2 mouse models.

Strengths:

The author achieved high protein loading and transfection efficiency using PEG10 self-assembly while packaging tumor neoantigens inside for cancer immunotherapy. The author also enhanced the targeting effect towards dendritic cells by surface modification using CpG-ODN.

Weaknesses:

There were some minor issues but they have been resolved in the revision process. It would be great if the authors could compare this with commercially available treatments and other vaccines.

Discussion:

Since the ePAC vaccine particle functions as a delivery platform, it can be tailored to different tumors when packed with their specific tumor neoantigens. Thus, the ePAC platform can be potentially employed in a broad range of cancer vaccine therapies. It would be exciting to see this platform being developed for other major cancer types.

# Referee report: Reviewer #2 (Public review)

**DOI:** 10.7554/eLife.98579.3.sa2

Summary:

The authors provided a novel antigen delivery system which showed remarkable efficacy in transporting antigens to develop cancer therapeutic vaccine.

Strengths:

This manuscript was innovative, meaningful, and had a rich amount of data.

Comments on the revised version:

All my concerns have been addressed by the authors

# Referee report: Reviewer #3 (Public review)

**DOI:** 10.7554/eLife.98579.3.sa3

Summary:

The authors harnessed the potential of mammalian endogenous virus-like protein to encapsulate virus-like particles (VLPs), enabling the precise delivery of tumor neoantigens. Through meticulous optimization of the VLP component ratios, they achieved remarkable stability and efficiency in delivering these crucial payloads. Moreover, the incorporation of CpG-ODN further heightened the targeted delivery efficiency and immunogenicity of the VLPs, solidifying their role as a potent tumor vaccine. In a diverse array of tumor mouse models, this novel tumor vaccine, termed ePAC, exhibited profound efficacy in activating the murine immune system. This activation manifested through the stimulation of dendritic cells in lymph nodes, the generation of effector memory T cells within the spleen, and the infiltration of neoantigen-specific T cells into tumors, resulting in robust anti-tumor responses.

Strengths:

This study delivered tumor neoantigens using VLPs, pioneering a new method for neoantigen delivery. Additionally, the gag protein of VLP is derived from mammalian endogenous virus-like protein, which offer greater safety compared to virus-derived gag proteins, thereby presenting a strong potential for clinical translation. The study also utilized a humanized mouse model to further validate the vaccine's efficacy and safety. Therefore, the anti-tumor vaccine designed in this study possesses both innovation and practicality.

# Author comment: Author response

**DOI:** 10.7554/eLife.98579.3.sa4

**Authors:** Ruijing Tang, Luobin Guo, Tingyu Wei, Tingting Chen, Huan Yang, Honghao Ye, Fangzhou Lin, Yongyi Zeng, Haijun Yu, Zhixiong Cai, Xiaolong Liu

**Affiliations:** Mengchao Hepatobiliary Hospital Fuzhou China; Mengchao Hepatobiliary Hospital Fuzhou China; Mengchao Hepatobiliary Hospital Fuzhou China; Mengchao Hepatobiliary Hospital Fuzhou China; Mengchao Hepatobiliary Hospital Fuzhou China; Mengchao Hepatobiliary Hospital Fuzhou China; Mengchao Hepatobiliary Hospital Fuzhou China; Mengchao Hepatobiliary Hospital Fuzhou China; Shanghai Institute of Materia Medica Shanghai China; Mengchao Hepatobiliary Hospital Fuzhou China; Mengchao Hepatobiliary Hospital Fuzhou China

The following is the authors’ response to the original reviews.

**Reviewer #1 (Public Review):**

Tang et al present an important manuscript focused on endogenous virus-like particles (eVLP) for cancer vaccination with solid in vivo studies. The author designed eVLP with high protein loading and transfection efficiency by PEG10 self-assembling while packaging neoantigens inside for cancer immunotherapy. The eVLP was further modified with CpG-ODN for enhanced dendritic cell targeting. The final vaccine ePAC was proven to elicit strong immune stimulation with increased killing effect against tumor cells in 2 mouse models. Below are my specific comments:

Thanks very much to comment our work as “important”. We sincerely appreciate the extremely helpful comments from the reviewer to significantly improve the quality of our manuscript.

(1) The figures were well prepared with minor flaws, such as missed scale bars in Figures 4B, 4K, 5B, and 5C. The author should also add labels representing statistical analysis for Figures 3C, 3D, and 3E. In Figure 6G, the authors should label which cell type is the data for.

Thanks very much for the very suggestive comments. The scale bars and statistical analysis have been added in Figures 4B, 4K, 5B, 5C, 3C, 3D, and 3E. For Figure 6G, we have added “CD44+ CD62L- in CD8+ T cells” to explain the cell type.

(2) In Figure 3H, the antigen-presenting cells (APCs) increased significantly, but there was also a non-negligible 10% of APCs found in the control group, indicating some potential unwanted immune response; the authors need to explain this phenomenon or add a cytotoxic test on the normal liver or other cell lines for confirmation.

Thanks very much for this extremely helpful suggestion. The antigen-presenting cells (APCs) in Figure 3H were isolated from mouse bone marrow and then cultured in vitro for about 5 days with cytokine stimulation (IL-4 and GM-CSF). Due to the stimulation effects of IL-4 and GM-CSF, a small proportion of the APCs (~10%) was tending to mature (co-expressing CD80 and CD86) in the control group, as pointing out by the reviewer. Similarly, in Figure 3I, these 10% activated APCs can activate T cells in vitro and exhibit certain cytotoxicity. Since APCs must be induced and cultured in vitro before using in this experiment, the background cytotoxicity induced by cytokines is unavoidable, and this has been well documented in literatures.

(3) In Figure 3I, the ePAC seems to have a very similar effect on cytotoxic T-cell tumor killing compared to the peptides + CpG group. If the concentrations were also the same, based on that, questions will arise as to what is the benefit of using the compact vector other than just free peptide and CpG? Please explain and elaborate.

Thanks very much for the comment. In vitro experiments indeed demonstrated that peptides + CpG had the same T cell activating ability as ePAC, as pointing out by the reviewer. However, due to the instability of peptides and the lack of targeting, the efficiency of activating the immune system for peptides + CpG after subcutaneous injection is significantly lower than that for ePAC in vivo, as shown in Figure 3D and Figure 2A. Then, as expected, the antitumor efficacy induced by peptides alone + CpG is significantly lower than that induced by ePAC in Figure 5. We have provided a detailed description in “Results” section of “Antitumor effect of ePAC in subcutaneous HCC model” as follows: Furthermore, ePAC with the ability to target DCs and increased stability by encapsulating peptides, exhibited significantly higher tumor growth inhibition efficiency (p=0.0002) comparing with the eVLP + CpG-ODN treated group similar to the simple mixture of neoantigen peptides and adjuvant (Figures 5B and 5C). Meanwhile, the Kaplan-Meier analysis of tumor progression free survival (PFS) also clearly demonstrated the therapeutic advantages of our ePAC (p=0.0194, Figure 5B).

(4) In the animal experiment in Figures 4F to L, the activation effect of APCs was similar between ePAC and CpG-only groups with no significance, but when it comes to the HCC mouse model in Figure 5, the anti-tumor effect was significantly increased between ePAC and CpG-only group. The authors should explain the difference between these two results.

Thanks very much for the comment. Since PEG10 protein does not have an adjuvant effect, the adjuvant effect of ePAC mainly comes from the modified CpG. Therefore, although ePAC can effectively deliver tumor neoantigens, it does not have a significant advantage over free CpG in activating APCs. However, CpG only possesses the adjuvant effect and does not carry neoantigens. While it can promote the maturation of APCs, it cannot generate neoantigen-specific T cells. Consequently, the antitumor effect of CpG-only is much lower than that of ePAC in Figure 5.

**Reviewer #2 (Public Review):**

Summary:

The authors provided a novel antigen delivery system that showed remarkable efficacy in transporting antigens to develop cancer therapeutic vaccines.

Strengths:

This manuscript was innovative, meaningful, and had a rich amount of data.

Weaknesses:

There are still some issues that need to be addressed and clarified.

Thanks very much to comment our work as “innovative”. We sincerely appreciate the extremely helpful comments from the reviewer to significantly improve the quality of our manuscript, and the listed weaknesses have been all carefully addressed.

(1) The format of images and data should be unified. Specifically, as follows: a. The presentation of flow cytometry results; b, The color schemes for different groups of column diagrams.

Thanks very much. Following the reviewer’s comment, we have unified the format of all images and data as suggested.

(2) The P-value should be provided in Figures, including Figure 1F, 1H, 3C, 3D, and 3E.

Thanks very much. We have provided the corresponding P-values in Figure 1F, 1H, 3C, 3D, and 3E.

(3) The quality of Figure 1C was too low to support the conclusion. The author should provide higher-quality images with no obvious background fluorescent signal. Meanwhile, the fluorescent image results of "Egfp+VSVg" group were inconsistent with the flow cytometry data. Additionally, the reviewer recommends that the authors use a confocal microscope to repeat this experiment to obtain a more convincing result.

Thanks very much for this comment. Following the reviewer’s suggestion, we uniformly adjusted the original images in Figure 1C to reduce background interference and increase its quality. After eliminating background interference, the fluorescence image of the "Egfp+VSVg" group was consistent with the flow cytometry result.

(4) The survival situation of the mouse should be provided in Figure 5, Figure 6, and Figure 7 to support the superior tumor therapy effect of ePAC.

Thanks very much for the extremely helpful comment. Following the reviewer’s suggestion, we have added the progression free survival (PFS) of mice in Figure 5 and described this result in the “Results” section of “Antitumor effect of ePAC in subcutaneous HCC model” as follows: Meanwhile, the Kaplan-Meier analysis of tumor progression free survival (PFS) also clearly demonstrated the therapeutic advantages of our ePAC (p=0.0194, Figure 5B). For Figure 6 and Figure 7, to promptly detect the immune changes in the tumor microenvironment after vaccination, we were unable to conduct long-term observations on tumor-bearing mice, and therefore, we did not provide the survival curve. However, we monitored the tumor volume changes in real-time, which also can serve as an important measure for evaluating antitumor efficacy.

(5) To demonstrate that ePAC could trigger a strong immune response, the positive control group in Figure 4K should be added.

Thanks very much for this very helpful comment. Following the reviewer’s suggestion, the mouse anti-CD3 antibody was used as the positive control in vitro to activate splenic T cells for ELISPOT assay, and the corresponding results have been added in revised Figure 4K. To address this, we have provided a detailed description in “Figure legends” section of “Figure 4. ePAC delivery and immune activation in vivo” as follows: The mouse anti-CD3 antibody was used to activate splenic T cells in vitro as the positive control for ELISPOT assay.

(6) In Figure 6G-I and other figures, the author should indicate the time point of detection. Meanwhile, there was no explanation for the different numbers of mice in Figure 6G-I. If the mouse was absent due to death, it may be necessary to advance the detection time to obtain a more convincing result.

Thanks very much for the comment. The samples for Figure 6 G-I data were collected and analyzed at the day 28 after the start of treatment. Following the reviewer’s suggestion, we have specifically marked the time point of “Sacrifice for sampling” in Figure 6A. And we have provided a detailed description in “Figure legends” section of “Figure 6. Evaluation ePAC antitumor efficacy in orthotopic HCC model by αTIM-3 combination” as follows: The mice were sacrificed and sampled for analysis on the day of 28 after initiating treatment. In addition, in Figure 6G-I we have clearly indicated the sample size for each group. Although three mice in the PBS group died, we still have obtained enough samples for statistical analysis (n>3).

(7) In Figure 6B, the rainbow color bar with an accurate number of maximum and minimum fluorescence intensity should be provided. In addition, the corresponding fluorescence intensity in Figure 6B should be noted.

Thanks very much for this very helpful comment. Following the reviewer’s suggestion, we have added the rainbow color bar with an accurate number of maximum and minimum fluorescence intensity, and the statistic results in revised Figure 6B.

(8) The quality of images in Figure 1D and Figure S1B could not support the author's conclusion; please provide higher-quality images.

Thanks very much. In Figure 1D and Figure S1B, to ensure the authenticity of the results, we tried our best to improve the quality of the pictures and provided the WB results with the full membrane scan. Although some non-specific bands appeared in the results, the target bands remained prominent. Additionally, we used two tags (HA and eGFP) for verification, which fully guarantees the reliability of our findings.

(9) In Figure 2F, the bright field in the overlay photo may disturb the observation. Meanwhile, the scale bar should be provided in enlarged images.

Thanks very much. Following the reviewer’s suggestion, we have deleted the bright field in revised Figure 2F and added the scale bar in the enlarged images.

**Reviewer #3 (Public Review):**

Summary:

The authors harnessed the potential of mammalian endogenous virus-like proteins to encapsulate virus-like particles (VLPs), enabling the precise delivery of tumor neoantigens. Through meticulous optimization of the VLP component ratios, they achieved remarkable stability and efficiency in delivering these crucial payloads. Moreover, the incorporation of CpG-ODN further heightened the targeted delivery efficiency and immunogenicity of the VLPs, solidifying their role as a potent tumor vaccine. In a diverse array of tumor mouse models, this novel tumor vaccine, termed ePAC, exhibited profound efficacy in activating the murine immune system. This activation manifested through the stimulation of dendritic cells in lymph nodes, the generation of effector memory T cells within the spleen, and the infiltration of neoantigen-specific T cells into tumors, resulting in robust anti-tumor responses.

Strengths:

This study delivered tumor neoantigens using VLPs, pioneering a new method for neoantigen delivery. Additionally, the gag protein of VLP is derived from mammalian endogenous virus-like protein, which offers greater safety compared to virus-derived gag proteins, thereby presenting a strong potential for clinical translation. The study also utilized a humanized mouse model to further validate the vaccine's efficacy and safety. Therefore, the anti-tumor vaccine designed in this study possesses both innovation and practicality.

Thanks very much to comment our work as “novel”, “innovation” and “practicality”. We sincerely appreciate the extremely helpful comments from the reviewer to significantly improve the quality of our manuscript.

Weaknesses:

(1) CpG-ODN is an FDA-approved adjuvant with various sequence structures. Why was CpG-ODN 1826 directly chosen in this study instead of other types of CpG-ODN? Additionally, how does DEC-205 recognize CpG-ODN 1826, and can DEC-205 recognize other types of CpG-ODN?

Thanks very much for the comment. CpG-ODNs are classified into three main types based on their structural composition: A, B, and C. Among them, only the B-class CpG-ODNs 1668, 1826, and 2006 have been directly proven to effectively bind DEC-205 and activate DC cells [1]. Therefore, in this study, B-class CpG-ODN 1826 was chosen as the ligand targeting DEC-205 on the surface of DC cells. DEC-205 primarily binds sequences containing the CpG motif core in a pH-dependent manner, thus theoretically allowing DEC-205 to bind a wide range of CpG-ODNs.

[1] Lahoud MH et al. DEC-205 is a cell surface receptor for CpG oligonucleotides. PNAS. 2012

(2) Why was it necessary to treat DCs with virus-like particles three times during the in vitro activation of T cells? Can this in vitro activation method effectively obtain neoantigen-responsive T cells?

Thanks very much for the comment. DCs need to be pre-stimulated before being used to activate T cells. Although Single DC stimulation can activate T cells, but the activation efficiency is insufficient. Current research suggests that three DC-T interactions can more effectively activate T cells [2]. Therefore, we prepared virus-like particle stimulated DCs for three times to fully activate T cells. Our results in Figures 3I and 7D also demonstrate that three-time stimulations effectively activated antigen-specific T cells, resulting in stronger tumor cell killing effects.

[2] Ali M et al. Induction of neoantigen-reactive T cells from healthy donors. Nature protocol. 2019.

(3) In the humanized mouse model, the authors used Hepa1-6 cells to construct the tumor model. To achieve the vaccine's anti-tumor function, these Hepa1-6 cells were additionally engineered to express HLA-A0201. However, in the in vitro experiments, the authors used the HepG2 cell line, which naturally expresses HLA-A0201. Why did the authors not continue to use HepG2 cells to construct the tumor model, instead of Hepa1-6 cells?

Thanks very much for the comment. HepG2 cells are derived from human liver cancer. When directly implant into immunocompetent mice, they will be cleared by the mouse immune system and will not form tumors. Therefore, we have not continued to use HepG2 cells to construct the tumor model.

(4) The advantages of low immunogenicity viruses as vaccines compared with conventional adenovirus and lentivirus, etc. should be discussed.

Thanks very much for the very suggestive comment. In the introduction starting from line 76, we first described the structure and function of lentiviruses and discussed the design and application of virus-like particles (VLPs) based on lentiviruses. To provide a more comprehensive comparison, we included a discussion on VLPs, lentiviruses, and adenoviruses in the discuss section (from line 441 to line 447) as follows: “Furthermore, comparing to the virus-based delivery vectors, the lentiviruses although can stably integrate into the host genome but carry risks of insertional mutagenesis; adenoviruses although have high transduction efficiency but strong immunogenicity, which leads to fast clearance by the immune system of the host and affects the efficiency of the secondary injection. Instead, our VLPs offer low immunogenicity and superior safety, making them more suitable for repeated use and vaccine development.”

(5) In Figure 6B, the authors should provide statistical results.

Thanks very much. We have provided the statistical results in revised Figure 6B following the reviewer’s suggestion.

(6) The entire article demonstrates a clear logical structure and substantial content in its writing. However, there are still some minor errors, such as the misspelling of "Spleenic" in Figure 3B, and the sentence from line 234 should be revised.

Thanks very much. We have carefully checked and corrected the typos throughout the whole manuscript as much as possible.

(7) The authors demonstrated the efficiency of CpG-ODN membrane modification by varying the concentration of DBCO, ultimately determining the optimal modification scheme for eVLP as 3.5 nmol of DBCO. However, in Figure 2B, the author did not provide the modification efficiency when the DBCO concentration is lower than 3.5 nmol. These results should be provided.

Thanks very much for the suggestion. We have repeated the experiment and reduced the concentration of DBCO to 2.1 nmol and 0.7 nmol, respectively. The results showed that in a 200 µl eVLP reaction system, 3.5 nmol DBCO achieved the highest modification efficiency. We have provided a detailed description in “Results” section of “Envelope decoration of neoantigen-loaded eVLP” as follows: Furthermore, by varying the concentration of DBCO-C6-NHS Ester from 0 to 14 nmol, ePAC exhibited different CpG-ODN loading efficiency as evidenced by agarose gel electrophoresis (Figure 2B and Figure S3). And the results showed that in a 200 µl eVLP reaction system, 3.5 nmol DBCO achieved the highest modification efficiency.

(8) In Figure 3, the authors presented a series of data demonstrating that ePAC can activate mouse DC2.4 cells and BMDCs in vitro. However, in Figure 7, there is no evidence showing whether human DC cells can be activated by ePAC in vitro. This data should be provided.

Thanks very much for this very helpful suggestion. We used ePAC to activate human DCs and the results indicate that, compared to the blank control group, both eVLP and ePAC increased the co-expression of CD80 and CD86 in DCs, and ePAC was the most efficient. We have provided a detailed description in the “Results” section of “Antitumor effect by HLA-A*0201 restricted vaccine” as follows: After the stimulation, the DCs in ePAC treated group showed the highest level of maturation comparing to the eVLP treated group and control group (Figure S4), by using flow cytometry analysis.”
